# A potential area of use for immune checkpoint inhibitors: Targeting bone marrow microenvironment in acute myeloid leukemia

**DOI:** 10.3389/fimmu.2023.1108200

**Published:** 2023-01-20

**Authors:** Başak Aru, Cemil Pehlivanoğlu, Zeynep Dal, Nida Nur Dereli-Çalışkan, Ege Gürlü, Gülderen Yanıkkaya-Demirel

**Affiliations:** ^1^ Immunology Department, Faculty of Medicine, Yeditepe University, Istanbul, Türkiye; ^2^ School of Medicine, Yeditepe University, Istanbul, Türkiye

**Keywords:** bone marrow microenvironment, immune checkpoint inhibitors (ICI), immune checkpoint proteins (ICP), acute myeloid leukemia, tumor microenvironment

## Abstract

Acute myeloid leukemia (AML) arises from the cells of myeloid lineage and is the most frequent leukemia type in adulthood accounting for about 80% of all cases. The most common treatment strategy for the treatment of AML includes chemotherapy, in rare cases radiotherapy and stem cell and bone marrow transplantation are considered. Immune checkpoint proteins involve in the negative regulation of immune cells, leading to an escape from immune surveillance, in turn, causing failure of tumor cell elimination. Immune checkpoint inhibitors (ICIs) target the negative regulation of the immune cells and support the immune system in terms of anti-tumor immunity. Bone marrow microenvironment (BMM) bears various blood cell lineages and the interactions between these lineages and the noncellular components of BMM are considered important for AML development and progression. Administration of ICIs for the AML treatment may be a promising option by regulating BMM. In this review, we summarize the current treatment options in AML treatment and discuss the possible application of ICIs in AML treatment from the perspective of the regulation of BMM.

## 1 Acute myeloid leukemia

Acute myeloid leukemia (AML) stems from the myeloid cell lineage and is defined as the presence of immature myeloid precursors (blast cells) in bone marrow or peripheral blood (1). Although it mostly affects adults, its clinical presentation and features vary among individuals. The latest WHO classification considers AML in 25 subtypes. Even though AML is mostly seen in blood and bone marrow, extramedullary manifestations can also be seen with certain types. AML manifests with aggressive progression, with an overall 5-year survival rate of approximately 25% (2, 3).

In AML, nonfunctional abnormally proliferated blast cells dominate the bone marrow and thus impair normal hematopoiesis that may result in pancytopenia which will further demonstrate itself with manifestations such as anemia, clotting disorders, and immunosuppression, where the latter increases vulnerability to infections (4–6). In some cases, exceedingly high leukocyte count can increase the risk of disseminated intravascular coagulation and leukostasis of which the latter leads to lethal manifestations related to the central nervous system (CNS) and lungs (7, 8). Patients may experience weakness, fatigue, pulmonary leukostasis and some abnormal bleeding, along with bruising resulting from minor traumas (9–11). Coagulation disorders are considered the most severe presentations of AML and they account for death in 7% of all cases (12).

Diagnosis of AML is made by the presence of 20% blast count in peripheral blood or bone marrow aspirate. Subtypes of the disease is assessed by flow cytometry to define the subtype of the disease, while chromosomal alterations are investigated using cytogenetic approaches, morphological changes in cells can be observed by bone marrow smears, and oncogenic mutations can be detected by genomic sequencing (1, 13).

## 2 Current treatment strategies in acute myeloid leukemia

Treatment strategies in AML depend on prior toxic exposure, precursor myelodysplasia, karyotypic and molecular abnormalities and patient-specific factors, including assessment of comorbid conditions, age, risk status, or disease situation such as relapsed or refractory. National Comprehensive Cancer Network (NCCN) Clinical Practice Guidelines in Oncology offer annually updated recommendations for the diagnosis and treatment of AML in adults, based on the reviews of recently published clinical trials which have led to significant improvements in treatment. Although details of treatment strategies are not a focus of this review, we will summarize the current therapeutic opportunities to provide a general perspective based on the NHHC 2022 guidelines (14, 15).

The European LeukemiaNet (ELN) risk stratification and the National Comprehensive Cancer Network (NCCN) guidelines classify AML patients into three groups that are associated with specific prognoses and may guide medical decision-making: favorable, intermediate, and poor (16). The classification is based on chromosomal and genetic abnormalities that certainly may have therapeutic significance, and likely to be changed as newer strategies become available. These markers include nucleophosmin 1 (NPM1), FLT3, CCAAT/enhancer-binding protein alpha (CEBPA), IDH1/2, DNA (cytosine-5)-methyltransferase 3A (DNMT3A), KIT, tumor suppressor protein 53 (TP53), Runt-related transcription factor 1 (RUNX1), and additional sex combs like-1 (ASXL1) gene mutations. FLT3 inhibitors (midostaurin, gilteritinib, quizartinib) are effective against FLT3-mutated AML, while IDH inhibitors (ivosidenib, enasidenib) are active against IDH1 or IDH2 mutated AML, respectively, and TP53 inhibitors (eprenetapopt) are effective against secondary AML and therapy-related leukemia. Other targeted therapy options include B-cell lymphoma 2 (Bcl2) inhibitors such as venetoclax; Hedgehog signaling pathway inhibitors such as glasdegib and hypomethylating agents (HMAs: azacytidine, decitabine) (17). Some patients admitted with isolated extramedullary disease may be eligible for systemic radiation therapy. In rare cases, local radiotherapy or surgery may be used for residual disease (18).

Currently, the main treatment for most types of AML is cytotoxic chemotherapy that consists of two phases: remission induction and post-remission consolidation treatments. Although patients are managed according to the same general therapeutic principles, chemotherapy regimens may vary depending on whether the patient is a candidate for intensive or non-intensive therapeutic regimens. In patients eligible for high intensity induction chemotherapy, the “7+3 regimen” of cytarabine plus anthracycline is commonly used (19). Other alternatives include fludarabine + cytarabine + granulocyte colony-stimulating factor + andidarubicin regimens (FLAG-IDA) and mitoxantrone-based cytarabine regimens (20). In addition to these regimens, addition of the kinase inhibitor midostaurin to induction therapy for FLT3-mutant AML patients has become standard (21). For remission consolidation therapies, regimens containing moderate doses of cytarabine are widely used and may improve blood count recovery. Despite the lack of a consensus, in patients who are not considered candidates for intensive therapy, following regimens are often used in the context of clinical trials: Azacitidine or decitabine + venetoclax combination, low dose cytarabine + venetoclax combination, azacitidine + ivosidenib combination (AML with IDH1 mutation), ivosidenib monotherapy for very frail patients (AML with IDH1 mutation) or best supportive care including hydroxyurea for patients who cannot tolerate or refuse any anti-leukemic therapy (20). To be considered in remission, bone marrow biopsy should show normocellular bone marrow while blasts should not exceed 5%; yet many patients develop relapsed and refractory diseases despite therapeutic options (22). Stem cell transplantations are reported to decrease the risk of leukemia relapse more than the standard chemotherapeutic approaches, yet they are also likely to lead to severe complications (23). Another approach in AML treatment is the administration of high doses of chemotherapeutics followed by either an allogeneic or autologous hematopoietic stem cell transplantation (HSCT). Currently, HSCT is the most recognized and frequently used cellular therapeutic option (24).

Antibody–Drug Conjugates (ADC), monoclonal antibodies that are linked to cytotoxic agents are novel treatment options in AML (25). The antibody targets a cell surface antigen that is exclusively expressed on tumor cells, the linker provides stability and enable selective intracellular release, and cytotoxic compound exerts DNA-damaging or microtubule-inhibitory activities (26). Contrary to conventional monoclonal antibodies, antibody conjugates in ADC do not induce any biological response. These antibodies should remain intact in the circulation, they have high target affinity while exerting limited immunogenicity and cross-reactivity. In 2017, Gemtuzumab ozogamicin (GO) became the first clinically approved ADC for the treatment of CD33-positive AML, and remains as the only FDA approved ADC for AML treatment (26). IMGN632 which combines an anti-CD123 antibody with a unique DNA-alkylating agent is another ADC that revealed promising results when tested in cell lines and animal models of AML as well as primary patient samples, and currently being tested in AML treatment either as monotherapy or in combination with venetoclax and/or azacytidine (27, 28).

Besides the abovementioned therapeutic interventions, other immunotherapeutic strategies in AML include immune checkpoint blockade, bispecific T cell engagers (BiTE), chimeric antigen receptor T cells (CAR-T) and tissue infiltrating lymphocytes (TIL) are under investigation (19, 22). As extensively described in the literature, the expression of inhibitory checkpoint proteins on AML blasts has been recognized as an important immune escape mechanism (29). Immune checkpoint inhibitors are under investigation for treatment of AML in many experimental and clinical studies.

## 3 Immune checkpoint inhibitors in treatment of acute myeloid leukemia

Immune checkpoints are receptor-based signal cascades that lead to negative regulation of immune cells, enabling escape from immune surveillance that eventually results in failure of tumor cell elimination favoring tumor progression. Immune checkpoint blockade exerts its’ anti-cancer effect by promoting the immune response through administration of monoclonal antibodies that target immune checkpoint proteins present on immune cells or tumor cells. Inhibition of immune checkpoints such as cytotoxic T lymphocyte antigen 4 (CTLA-4), programmed death-1 (PD-1), and programmed death-ligand 1 (PD-L1) enhances immune responses by inhibiting negative signaling receptors and supporting immune activation, where, in turn, elimination of the tumor promotes cancer regression. Currently, three different classes of Immune Checkpoint Inhibitors (ICIs); PD-1 inhibitors (cemiplimab, nivolumab, pembrolizumab, dostarlimab), PD-L1 inhibitors (avelumab, atezolimumab, durvalumab), and one CTLA-4 inhibitor (ipilimumab) have been approved by the US Food and Drug Administration (FDA) for the treatment of various malignancies while others targeting T cell immunoglobulin and mucin domain 3 (TIM3) and lymphocyte activating-3 (LAG-3) are still under investigation (30–32). All checkpoint pathways differ from each other according to the stages they involve the in the immune responses as well as their signaling mechanisms; however, the common purpose of ICIs is to observe similar impact on T-cell activity and clinical regression of cancer.

Although ICIs are already being used in the treatment of various malignancies, studies on AML are still ongoing. There are many completed and ongoing experimental studies and clinical trials in distinct phases evaluating ICIs in treatment of AML either as monotherapy or part of a combinational therapy with other agents including chemotherapeutics, HMAs or other immunotherapies. Experimental studies and clinical trials regarding ICIs, either in combination with other therapeutic interventions or alone are summarized below.

### 3.1 Experimental studies on immune checkpoint inhibitor therapy in acute myeloid leukemia

Importance of IC pathways in immune evasion of AML as well as their blockade with specific agents in AML treatment has been underlined in several experimental studies which involved AML cell lines and murine models.

Constitutive expression of regulatory cell surface antigen CTLA-4 expression in more than 80% of AML samples was first reported two decades ago (33) and in 2006, its’ blockade with monoclonal antibodies were reported to enhance T cell responses in AML *in-vitro* (34). In a study involving a DA1-3b mouse model of AML, leukemic cells were reported to be present months despite the presence of an effective antileukemic immune response. Persistent leukemic cells were reported to have enhanced B7-H1 (PD-L1) and B7.1 expressions and resistant to cytotoxic T cell (CTL) mediated killing (35). The authors stated that an effective immunotherapeutic intervention should facilitate leukemia rejection and targeting overcoming the mechanisms that lie behind tumor dormancy and revealed that inhibition of B7-H1 (PD-L1) and B7.1/CTLA-4 interactions augmented CTL-mediated killing of the persistent cells as well as prolonging survival of naive mice injected with persistent leukemic cells. However, it should be noted that targeting B7.1/CTLA-4 and PD-1/PD-L1 axes may target different mechanisms compared to monotherapies (36), and elucidating such pathways in leukemias may pave the way for novel combinatorial therapies.

In terms of PD-1/PD-L1 axis, numerous experiments revealed upregulated expressions of both proteins in murine leukemia cells while demonstrating that genetic ablation or pharmacological inhibition of PD-1 can suppress leukemic cell proliferation and enhance survival in AML bearing mice (37). Combinatorial administration of innate immune agonists along with an ICI has revealed promising results by enhancing anti-tumor activity in a preclinical AML model: an innate immune agonist 5,6-dimethylxanthenone-4-acetic acid (DMXAA) activated the stimulator of interferon genes (STING) pathway that promoted dendritic cell maturation and in turn, maturation of leukemia-specific T-cells, resulting in a prolonged overall survival in leukemic mice (38). In anti-tumor responses, type I interferons (IFN) promotes the infiltration of CD8+ T cells, hence acts as a bridge between the innate and adaptive immunity (39, 40). This pathway also activates STAT6 and nuclear factor kappa B (NF-κB) pathways that result in the production of inflammatory mediators including TNF-α, IL-6 and CCL2/MCP-1 (41, 42). Unlike solid tumors, type I IFN response is shown not to be activated in hematological malignancies and activating STING pathway to promote anti-leukemic T cell responses stands out as a promising strategy (43). However, expression of immunosuppressive indolamine-2,3-dioxygenase (IDO) and upregulation of PD-L1 as a response to IFN-γ may be the restricting factors for the administration of STING agonists as a single agent in AML treatment. Thus, determination and inhibition of immune escape pathways induced by STING activation may enable STING agonists’ administration in the clinical setting. In line with this hypothesis, DMXAA inhibited the growth of murine AML cell line C1498 and increased PD-L1 expression while combination of PD-1 inhibition along with DMXAA therapy boosted activated host T cell numbers and bone marrow PD-1/L1/L2 expression, reducing disease burden and prolonging overall survival *in-vivo* (44). In an *in-vitro* study, DMXAA exposure promoted PD-L1 expression while leading to a slight increase in apoptosis and IL-6 and IFN-b production in C1498 AML cell line while coupling DMXAA with an anti-PD-1 antibody significantly reduced disease burden and extended general survival in C1498 grafted leukemic mice (45). Mice receiving combinatorial treatment exhibited boosted memory T-cells and mature dendritic cells along with lesser numbers of regulatory T-cells, proving apoptosis of leukemic cells. These findings were further supported by increased serum levels of type I interferons (IFN) and IFN-γ. These studies suggest that STING agonists can be used in combination with ICI for enhanced anti-tumor efficacy. Besides DMXAA, other STING agonists include GSK3745417 that has been shown promising anti-cancer activity on AML cell lines as well as primary AML cell cultures and MIW815 (ADU-S100) which recently have been reported to induce systemic immune activation while being well tolerated in patients with advanced/metastatic cancers, though AML was not investigated in the latter (46, 47). A recent study revealed that a novel STING agonist SHR1032 enhanced anti-tumor immunity and induced AML apoptosis under *in-vitro* and *in-vivo* settings (48). Besides AML, STING agonists have been under evaluation for the treatment of other solid and hematological malignancies: Ulevostinag (MK-1454) has been tested in combination with pembrolizumab in participants with advanced/metastatic solid tumors or lymphomas (49), while GSK3745417 is currently being tested either alone or in combination with PD-1 inhibitor dostarlimab (50), and BMS-98630 is being tested alone or in combination with nivolumab and ipilimumab in patients with advanced solid tumors (51). However, there are certain questions to be addressed before the implementation of STING agonists in the field of immune oncology, including whether the overstimulation of the pathway can induce autoimmune conditions, or if the pathway is a valid target in case of epigenetic silencing of STING (52).

Recent findings suggest that AML cells express high levels of TIM-3 and release galectin-9 (Gal-9) that impair activity of cytotoxic T cells and NK cells (53). The association between PI3K/Akt/mTOR signaling pathways and the regulation of immune checkpoint ligands including PD-L1, Galectin-9 (Gal-9), and CD155 was investigated in human AML cell line HL-60 *in-vitro*. For this purpose, cells were treated with idelalisib as PI3K inhibitor, MK-2206 as Akt inhibitor, and everolimus as mTOR inhibitor either in a single or combined format (54). Combinatorial treatment of HL-60 cells with two or three inhibitors diminished the expression levels of PD-L1, Gal-9, and CD155 checkpoint ligands, decreased proliferation and enhanced apoptosis. This study revealed that besides their cytotoxic properties, drugs targeting the PI3K/Akt/mTOR pathway play role in the regulation of ICP expression and interfere with immune evasion mechanisms of AML cells.

Recently, Xu et al. reported co-expression of PD-1 along with TIGIT on CD8+ T cells of AML patients’ bone marrow samples, moreover PD-1 and TIGIT positivity on CD8+ T cells showed positive correlation with age, suggesting greater T cell dysfunction in elderly patients. This study also revealed the increased frequency of PD-1+ and TIGIT+ CD8+ T cells in bone marrow samples compared to peripheral blood, a finding that indicates the importance of targeting immunosuppressive bone marrow microenvironment (BMM) in AML treatment (55). In another study aiming to characterize NK cell subsets of AML patients in bone marrow and peripheral blood, Brauneck et al. revealed TIGIT and poliovirus receptor-related immunoglobulin domain-containing (PVRIG) co-expression on NK cells of AML patients, and their simultaneous blockade enhanced the NK cell mediated killing *in-vitro* (56). In another study, Li et al. reported PVRIG ligand (poliovirus receptor-related 2, PVRL2) on AML patient blasts, and proven that blocking the PVRIG/PVRL2 axis enhanced NK cell activation and in turn, promoted killing of patient derived primary AML blasts (57).

CD47 is a macrophage ICP that is particularly involved in myeloid malignancies and has been identified as a leukemic stem cell marker in AML. Blockade of CD47-SIRPα pathway has been shown to increase several therapeutics in pre-clinical studies (58). Similarly, CD200 plays role in the formation of T regulatory cells (Tregs) is commonly overexpressed in AML blasts and shown to be associated with poor outcome (59). Along with CD200 on AML blasts, TIM-3 expression on peripheral blood T cells was proven to be involved in AML development, and these proteins hold the potential to serve as prognostic markers (60).

Programmed Death-1 Homolog (V-domain Ig suppressor of T cell activation, VISTA) is a novel co-inhibitory molecule that promotes immune evasion in solid tumors, and an *in-vivo* study revealed the connection between PD-1H and epigenetic modifications as well as their role in immune evasion in AML where DNA methyl transferase inhibition by 5-aza-2’-deoxycytidine (Decitabine) increased T cell infiltration that potentiated the anti-leukemic effect of the PD-1H blockade and significantly prolonged survival (61). VISTA has also been shown to be expressed on myeloid-derived suppressor cells (MDSCs) present in the peripheral blood of AML patients and contribute to the inhibition of T cell responses in AML (62). Moreover, authors reported a positive correlation between VISTA expression on MDSCs and PD-1 expression on T cells of AML patients, highlighting the potential of combinatorial VISTA and PD-1 inhibition in leukemia treatment. In an *in-vitro* study, both CTLA-4 and LAG-3 expression levels were reported higher in comparison with healthy controls in AML, and the receiver-operating characteristic (ROC) curve analysis suggested that CTLA-4 and LAG-3 co-positivity can be used as a diagnostic criteria for the disease (63).

It should be noted that even if the ICIs are promising in the cancer treatment, the broadly distributed immune-related adverse events (irAEs) may not be tolerable in some cases. To overcome this, some experimental studies focus on restricted immune checkpoint blockade such as α-PD-1 × α-CD3 × α-CD33, a bifunctional checkpoint inhibitory T cell–engaging antibody (CiTE) that directs T-cells to CD33 on AML cells with locally restricted immune checkpoint blockade (64). By the synergistic effect of ICI and avidity-dependent binding, PD-1 attachment improved T-cell activation (3.3-fold elevation of IFN-γ) and led to efficient and highly selective cytotoxicity against CD33+ PD-L1+ cell lines as well as patient-derived AML cells. In a murine xenograft model, CiTE induced complete AML eradication without initial signs of irAEs.

### 3.2 Clinical trials on immune checkpoint inhibitor therapy in acute myeloid leukemia

HMAs have been approved by FDA, and they are being used as epigenetic modifiers for the treatment of myelodysplastic syndromes (MDS) and acute AML patients, who are not eligible for induction chemotherapy (65, 66). It is reported that in these patients who underwent the treatment with HMAs, surface expression of ICPs (PD-L1, PD-L2, PD-1, and to a lesser extent, CTLA-4) increased in a dose-dependent matter. For the patients for whom the up-regulation of PD-L1 was to the greatest extent, it is reported that the response to HMA therapy was the shortest, and it was associated with a lower survival (67). Concerning these observations, clinicians suggested that HMA therapies lead to immune checkpoint activation and up-regulation, indicating that this resistance may be overcome by combining HMA with ICIs (68, 69).

In a phase 2 clinical trial, nivolumab was administered with azacitidine to a high-risk population of relapsed or refractory (R/R) AML patients. Among 70 patients, the response rate to therapy was 33%, with 22% being in complete remission or incomplete hematologic recovery. Grade 3/4 irAEs were reported in 11% of patients, the most frequent one being pneumonitis. For all 70 patients, the median survival was 6.3 months, while for 32 salvage-1 patients (the first therapy administered after all standard treatments proved ineffective), it was 10.5 months. This finding indicates a promising response rate for the combination therapy, as also stated by the authors. A greater response rate was recorded in patients with higher CD3+CD8+ T cell infiltration pre-therapy. Thus, it was reported that pre-therapy T cell infiltration can be considered an inflamed tumor marker and a biomarker that can be used in deciding which patient group would benefit from ICI-based treatments (70). In an expanded cohort study as a follow-up study to the clinical trial, the anti-CTLA-4 antibody ipilimumab was added to azacitidine and nivolumab regimen and administered to 24 R/R AML patients. The study has reported a 1-year overall survival of 45% in R/R AML patients. When this new triple combination treatment is compared in the aspect of the median overall survival, with the previous azacytidine and nivolumab double treatment and with the current treatment with hypomethylating agents, the results were respectively 10.5, 6.4 and 4.6 months. These findings demonstrate an encouraging and promising efficacy. Although regarding its safety, it is worth mentioning that in 6 patients (25%), grade 3/4 immune-related toxicity, including rash, pneumonitis, and colitis was reported (71). In another phase 2 study, the anti-PD-1 antibody pembrolizumab was administered to recently diagnosed R/R AML patients in combination with azacitidine. In this cohort, out of 29 eligible patients, 4 (14%) achieved complete remission or incomplete hematologic recovery, while 1 patient (4%) had partial remission. The median overall survival was 10.8 months. After 22 newly diagnosed older AML patients not eligible for intensive chemotherapy joined the study, out of 17 of whom were evaluable, 47% achieved complete remission or incomplete hematologic recovery, while 12% had partial remission. The new median survival was 13.1 months. These two cohorts display that azacitidine and pembrolizumab combination therapy proved beneficial in both R/R and recently diagnosed older patient groups. Grade 3/4 irAEs were observed in both patient groups, the ratios being more frequent (24%) in the first cohort and less (14%) in the second. Although at this point this treatment combination looks more suitable to newly diagnosed older patient groups, more specifically directed research is still needed (72).

A phase 1b/2 study reported that azacytidine leads to PD-1 and PD-L1 upregulation in AML which causes drug resistance that may be overcome by including the PD-1 inhibitor nivolumab. In a study, azacitidine was combined with nivolumab and administered to 35 relapsed AML patients. Out of 35 patients evaluated, the preliminary data from this study showed that 6 (18%) were in complete remission (CR) or CR with incomplete count recovery (CRi) and 5 (15%) were in hematologic improvement. A decrease in blast count greater than 50% was observed in 14 patients (26%), and the median overall survival was reported as 9.3 months (range, 1.8 - 14.3). Patients with CR/CRi, higher levels of pre-treatment CD3+ and CD8+T-cell infiltration were detected in bone marrow aspirates (73). In conclusion, azacytidine in combination with nivolumab yielded a promising and durable response in relapsed AML, and irAEs may be managed with systemic steroid administration.

In another multi-centered, randomized, international phase 2 clinical trial, azacitidine was administered to high-risk MDS or AML patients in combination with the anti-PD-L1 antibody durvalumab or as a single agent. In this study with 129 AML patients older than 65 years old who were not eligible for chemotherapy; a comparison between the azacitidine and Durvalumab combination therapy and azacitidine as a single agent therapy showed no statistically significant difference in total response rate (31.3% vs. 35.4%) or complete remission rate (17.2% vs. 21.5%). The overall survival rate was 13.0 and 14.4 months, respectively, with no unexpected side effects. Although this study portrays an important role regarding its comparatively larger sample size, it is worth considering that more than half of the patients did not continue with the study regimens, which might be taken into account in interpreting the results (74).

The resistance mechanisms and biomarkers playing role in processes that play role in treatment response are not yet fully explained, but a study from Herbrich et al. puts forth a possible explanation. In their study, Herbrich et al. evaluated the bone marrow and peripheral blood samples taken from nine relapsed or refractory AML patients who received azacitidine and anti-PD-L1 antibody avelumab using single-cell mass cytometry. Out of nine evaluable patients, four had an initial decrease in blast count, and seven showed a fast progression subsequently. Authors reported that in AML bone marrow, CD4+ and CD8+ T cells had a significantly lower proportion of naïve T cells at baseline, along with a smaller ratio of terminally differentiated CD8+ cells. Contrarily, the largest portion of T-cells in AML bone marrow consisted of the effector memory CD4 and CD8 cells. In these patients, a high PD-L2 protein expression was observed in AML cells, and PD-L1 expression was low in the samples taken at both baseline and during therapy. PD-L2 was also frequently expressed in the newly formed clones which were not present at baseline. These findings may indicate a possible explanation for the different response rates to PD-1 and PD-L1 inhibition observed during AML treatment. These findings also indicate that the immune cell distribution is significantly affected in AML patients’ bone marrow. The T cell distribution ratio and the different checkpoints that are expressed on AML cells, such as PD-L2, may pose a key in the consideration of the approach and response of the treatment (75, 76).

In their study, Berger et al. administered anti-PD-1 monoclonal antibody CT-011 (pidilizumab) to patients with advanced hematologic malignities in a phase 1 clinical trial, where pidilizumab was administered to 17 patients (8 being diagnosed with AML) in doses between 0.2 and 0.6 mg/kg. Complete remission was observed in one patient, while clinical benefit is reported in 33%. Although serious adverse events were reported in 4 patients, who were all diagnosed with AML and passed away later, the study reported that none of these were related to the treatment but to fulminate-resistant leukemia and that the dose aforementioned can be considered safe (77, 78). Currently, pidilizumab is also being investigated in combination with a dendritic cell vaccine on AML patients in complete remission (79). In a phase 1/1b multi-centered study performed with hematologic cancer patients who were in relapse following post-allogenic HSCT, the anti-CTLA-4 antibody ipilimumab was administered to the patients. In 22 patients who were receiving 10 mg/kg, four were diagnosed with extramedullary AML, and one was diagnosed with MDS which progressed to AML; five patients (23%) were in complete remission, 2 (9%) showed partial response and 6 (27%) had a decreased tumor burden. A sustained response for longer than a year was reported in four patients. Although this study was noted to be attainable in patients with AML post-allogenic HSCT; the irAEs were reported in 6 patients (21%) including one death reported. Graft versus host disease (GvHD) is also reported in 4 (14%), which resulted in the conclusion of further application of ipilimumab. Altogether, these data revealed promising results regarding ipilimumab administration in patients with post-allogenic HSCT relapsed AML (65, 80). In a phase 2 study evaluating the effect of PD-1 inhibition after cytotoxic chemotherapy on clinical response, 37 patients diagnosed with relapsed or refractory AML were administered high-dose cytarabine followed by IV 200 mg pembrolizumab on the 14^th^ day. The patients who responded to the treatment continued to receive pembrolizumab for two years. The overall response rate was 46%, the composite complete remission was 38%, and the median overall survival was 11.1 months. For refractory or early relapsed patients, and for patients who received the treatment as the first salvage, the median overall survival was 13.2 and 11.3 months, respectively, which was considered promising by the authors. Grade 3 and higher irAEs were reported to be rare and self-limiting, with 14% which is promising when treatment feasibility is considered (81). In another phase 2 study, a patient group of nine who received pembrolizumab following high dose cytarabine was compared with a control group of 18 who underwent allogeneic HSCT and didn’t receive ICI. According to the one-year survival data, no significant difference was reported between the two groups (67% vs. 78%; p=0.34). For the group that received ICI, the 100-day mortality rate was 0%, while in the control group, it was 17%. Grade 3/4 acute GVHD risk didn’t increase in patients who received pembrolizumab prior to allogeneic HSCT while no indicator of chronic GVHD was reported (82). These findings support the aforementioned phase 2 study, in reflecting both the clinical activity and safety profile of cytarabine and pembrolizumab combination. Besides agents targeting PD-1/PD-L1 axis, anti-leukemic potential of humanized anti-TIM-3 antibody sabatolimab in combination with HMAs was investigated in 48 patients who were newly diagnosed with AML and ineligible for intensive chemotherapy (83). The overall response rate was reported as 40% while 30% of these patients achieved CR/CRi.

When ICIs’ role in maintenance is considered, preclinical studies indicate that ICIs can prevent leukemic cells’ evasion of the immune system and, thus, overcome tumor persistence. Another phase 2 study investigating efficacy of nivolumab on 14 high-risk AML patients in complete remission who were not eligible for allogeneic HSCT indicated that by the end of the year, 71% of patients were in complete remission, indicating the drug’s safety and feasibility in high-risk AML (84).

In brief, numerous recent clinical studies involving ICI as a single agent or combined with other treatments demonstrated promising results regarding clinical efficacy and safety profile. However, it is early to draw distinct conclusions about ICIs’ use in AML and further research is needed. It should be noted that currently, there is no ICI approved by the FDA in the treatment of AML, and the clinical trials regarding ICI in AML treatment are still at the early stages with results revealing modest efficacy, especially for monotherapy the refractory settings (85).

As mentioned earlier, chemotherapy in AML is divided into two phases; induction therapy and consolidation therapies which both vary according to the patient’s age, presence of co-morbidities and genetic alterations. Induction therapy aims to eliminate the blasts in the peripheral blood and to restore normal hematopoiesis while consolidation therapy is administered to remove residual leukemic cells (86). In clinical trials, efficacy of ICIs is mainly investigated in combination with chemotherapy agents and HMA (87). intervention in AML remains as allogeneic HSCT while the clinical trials involving ICI are ongoing and up-and-coming.

## 4 The interaction between bone marrow microenvironment and cancer cells in AML

**Figure 1:**
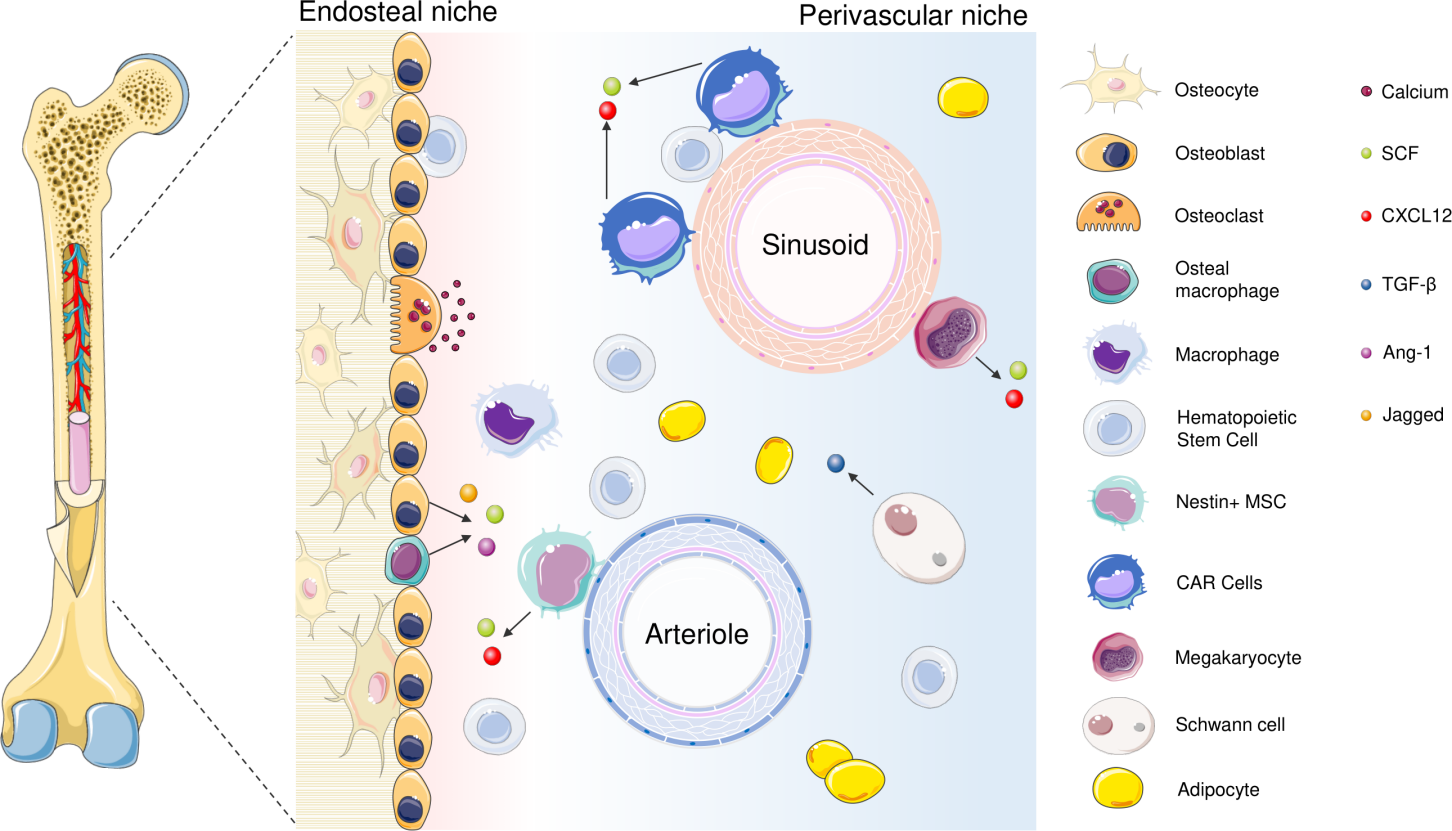
The cellular components of the BM niches include endothelial cells, HSCs, megakaryocytes, osteoblasts, osteoclasts, adipocytes, sympathetic neurons that are related to Schwann’s cells, bone macrophages and reticular cells (90, 92). Both soluble factors and direct contact between cells regulate HSC maintenance. Quiescent HSCs are kept in contact with osteoblasts and Nestin+ MSCs as well as CXCL12 - abundant reticular (CAR) cells in the perivascular niche; both secrete soluble factors such as Stem Cell Factor (SCF), CXCL12 (CXC motif chemokine ligand 12) and Ang-1 (Angiopoietin-1) while in the perivascular niche, secrete (93). Osteomacs, the bone-marrow-resident macrophages are also found in the endosteal niche and facilitate colonization; in the absence of osteomacs, HSCs are shown to leave BM and join circulation (92, 94). Jagged-1 is released from cells of osteocyte lineage, and responsible for the activation of Notch pathway (95). Organized as a monolayer in the internal compartment of blood vessels, ECs take part in various physiological processes including facilitating blood flow, contributing coagulation, nutrient exchange and regulate hematopoiesis (96). According to their localization in the BM, they are divided into two categories: sinusoid endothelial cells (SECs) which have low CD31 and Endomucin expression (type L), or arteriolar endothelial cells (AECs) with CD31 and Endomucin expression (type H). Both cell types play different roles in the modulation of BM niche (97). SECs are the compartments of more permeable sinusoidal vessels and secrete high levels of CXCL12 as well as E-selectin that regulate HSC homing (96, 98). On the contrary, AECs are the compartments of arteriolar vessels which have low penetration and ensure a relatively hypoxic environment (99, 100). AECs generate SCF which play a fundamental role in maintaining HSCs and express CXCL12. In addition, they produce Netrin-1 that retains HSCs’ quiescence and self-renewal (101). Megakaryocytes are the basic subunit in the perisinusoidal area and regulate HSC quiescence (102–104). In the endosteal niche, osteoblasts stabilize bone formation and produce mediators which are essential for HSC maintenance; and CXCL12, granulocyte colony-stimulating factor (G-CSF), SCF, Annexin 2 (ANXA2), Ang-1, Thrombopoietin (TPO) that are required for the regulation of HSC homing, quiescence and mobilization (105–113). Schwann cells are found in the perivascular niche and protect the quiescent HSCs through transforming growth factor-β (TGF-β) (114). Bone marrow adipocytes were also reported to support HSC proliferation through secreting adiponectin and contribute to energy metabolism (115, 116).

Bone marrow is an extraordinary tissue where various cells from lineages reside. BMM is a substantial gatekeeper in maintenance of the blood cell formation and is a complex structure which is composed of cellular and noncellular elements (88). The cellular elements consist of hematopoietic cells, stromal cells (fibroblasts, endothelial cells, endothelial progenitor cells, osteoblasts, osteoclasts, adipocytes) and noncellular elements consists of ECM components, autonomic nervous system and soluble factors such as cytokines (89). BMM is usually divided into two different anatomical locations as endosteal niche and perivascular niche (90); the main function of endosteal niche and perivascular niche is to aid long term storage of HSCs by providing a hypoxic environment and to support the proliferation and differentiation of HSCs by maintaining a more oxygenated environment, respectively. Based on their different functions and structural features, these niches have been divided into various subgroups; endosteal niche mainly comprise of osteo-lineage cells while the perivascular niche consists of different subtypes associated with endothelial and perivascular cells (91) (**Figure 1**). Various cellular or non-cellular components of BM is critical for maintenance of physiological conditions of microenvironments (92). In addition, in some sources, a third bone marrow niche called reticular niche, a transitional zone of endosteal and perivascular niches is described (117).

In leukemia, a growing body of evidence indicates leukemic cells’ involvement in malignant transformation, disease progression, treatment resistance, and relapse as the interplay between leukemic stem cells and the microenvironment alters the hemostasis in a way to support leukemic cells’ survival and proliferation, suggesting a bidirectional interaction between HSCs and BMM (118, 119). AML cells mainly bind to the BM fibroblast, fibronectin and laminin (120); SCF exposure enhances these cells’ adhesion to fibronectin (121). Both SCF and fibronectin are found in the BMM at high levels, and together they protect AML cells from apoptosis (121). These cells also remodel the BMM *via* secreting matrix metalloproteinases (MMPs) (122, 123). MMP-2 and -9 have been indicated to be secreted by leukemic blasts and involved in dissemination of myeloproliferative malignancies including AML. Thus, it can be concluded that the mediators released by the BMM play role in survival of the leukemic cells as well as regulating their mobilization, and in leukemia treatment, targeting BMM-related signaling pathways has been shown to enhance the therapeutic efficacy (124). Moreover, various BM-derived populations including myeloid cell–derived suppressor cells, mesenchymal stem cells, and tumor-associated macrophages are shown to be involved in escaping anti-tumor immune responses by suppressing anti-tumor responses (125). Angiogenesis enhances leukemogenesis by providing necessary factors that favor malignancy as certain angiogenic cytokines and factors were reported to be increased in AML patients and it was associated with poor prognosis (126). Lipolysis and remodeling of BMAT are induced in the setting of AML, and free fatty acids yielded by lipolysis are used as nutrient by leukemic cells (127). Sympathetic neuropathy may be seen due to bone marrow infiltration of malignant cells, and it was associated with AML progression (128). Along with chronic myeloid leukemia (CML), the niche microenvironment of acute myeloid leukemia (AML) is well established: with the help of recent studies, significant progress has been made in understanding the impact of genetic mutations or functional alterations in the BM on leukemia. Examples include the deletion of Dicer1 in osteoprogenitors, which leads to the development of myelodysplastic syndrome (MDS) with sporadic transformation to AML (129), or overexpression of β-catenin in osteoblasts as observed in 38% of the patients diagnosed with MDS or AML (130). Similarly, activation of the parathyroid hormone receptor in osteoblasts is shown to promote KMT2A-MLLT3 oncogene-induced AML (131). AML cells are also capable of modulating the BMM as cells harboring BCR-ABL1 and Nup98/HoxA9 fusion gene are indicated to inhibit mature osteoblasts and disrupt bone homeostasis by secreting CCL3 (132). Likewise, KMT2A-MLLT3 AML cells have been shown to inhibit terminal differentiation of bone marrow mesenchymal stromal cells to mature osteoblasts, which eventually results in decreased bone mineralization (128).

Recently, upregulated ICP expressions including PD-1, TIM-3, LAG-3 in addition to expansion of myeloid-derived suppressor cells and increased Treg frequency in the BMM of AML patients were reported, which highlights the importance of IC blockade as a novel therapeutic strategy in the treatment of the disease (133).

### 4.1 Targeting bone marrow microenvironment in acute myeloid leukemia – existing strategies

When considering treatments targeting BM microenvironment, CXCL12 (C-X-C motif chemokine ligand 12)/CXCR4 (C-X-C chemokine receptor 4) axis is the most studied pathway in AML treatment; as reported, inhibition of this pathway leads to mobilization of leukemic cells, sensitizes them to chemotherapy and promotes apoptosis (134–138). The anti-CXCR4 antibody ulocuplumab has shown to inhibit CXCL12-mediated cell migration and promote apoptosis in *in vivo* murine AML models as well as promoting chemosensitivity *via* mobilizing AML cells to circulation in clinical studies (139–141). Another common strategy is inhibiting the Wnt/β-catenin pathway to diminish the protection provided by BMM: β-catenin is highly expressed in unfavorable and relapsed AML patients, and Wnt/β-catenin inhibitor PRI-724 was shown to suppress cell growth while promoting apoptosis in AML blasts and stem/progenitors (142). Wnt/β-catenin/FLT3 inhibitor SKLB-677 promoted apoptosis in FLT3-driven AML both *in-vitro*, *in-vivo* and *exvivo* (143). Another Wnt/β-catenin inhibitor, BC2059 has shown promising results in treatment of AML stem or blast progenitor cells with FLT3 internal tandem duplication expression in combination with receptor tyrosine kinase inhibitors quizartinib and crenolanib (144).

Targeting adhesion molecules which support the leukemic cells is another approach in AML treatment. Being the receptor of vascular cell adhesion molecule (VCAM-1), integrin α4β1 (very late antigen 4 – VLA 4) plays role in the adhesion of leukemic myeloblasts to BMM (145). Humanized VLA-4 monoclonal antibody natalizumab has been reported to induce mobilization and sensitize leukemic cells to chemotherapy (146). In combination with cytarabine, VLA-4 inhibitor FNIII14 has shown to eradicate minimal residual disease in a murine AML model, underlining the importance of inhibiting cell adhesion-mediated drug resistance (147). By regulating VLA-4 avidity, adhesion molecule CD44 was shown to strengthen the connection between AML cells and BMM, thus, contributing to the supportive BMM (148). In a phase I study, humanized anti-CD44 monoclonal antibody RG7356 was found to be safe and well tolerated though it is not suitable as a monotherapy due to its’ limited clinical activity in AML treatment (149).

The endothelial cell adhesion molecule E-selectin is another important component of the vascular niche that regulates the balance between HSC renewal and commitment. However, the inflammatory mediators secreted by AML blasts increase the expression of endothelial niche E-selectin, which, in turn, promotes their survival and chemoresistance through AKT/NF-κB/mTOR signaling pathways (150). In an AML murine model bearing the human KMT2A-MLLT3 oncogene, the small molecule E-selectin mimetic GMI-1271/Uproleselan has enhanced the efficacy of AML treatment by overcoming vascular niche-mediated chemoresistance, indicating E-selectin blockade alleviates pro-survival signaling and improving therapeutic efficacy (150).

### 4.2 Targeting bone marrow microenvironment in acute myeloid leukemia with an emphasis on immune checkpoint proteins

**Figure 2:**
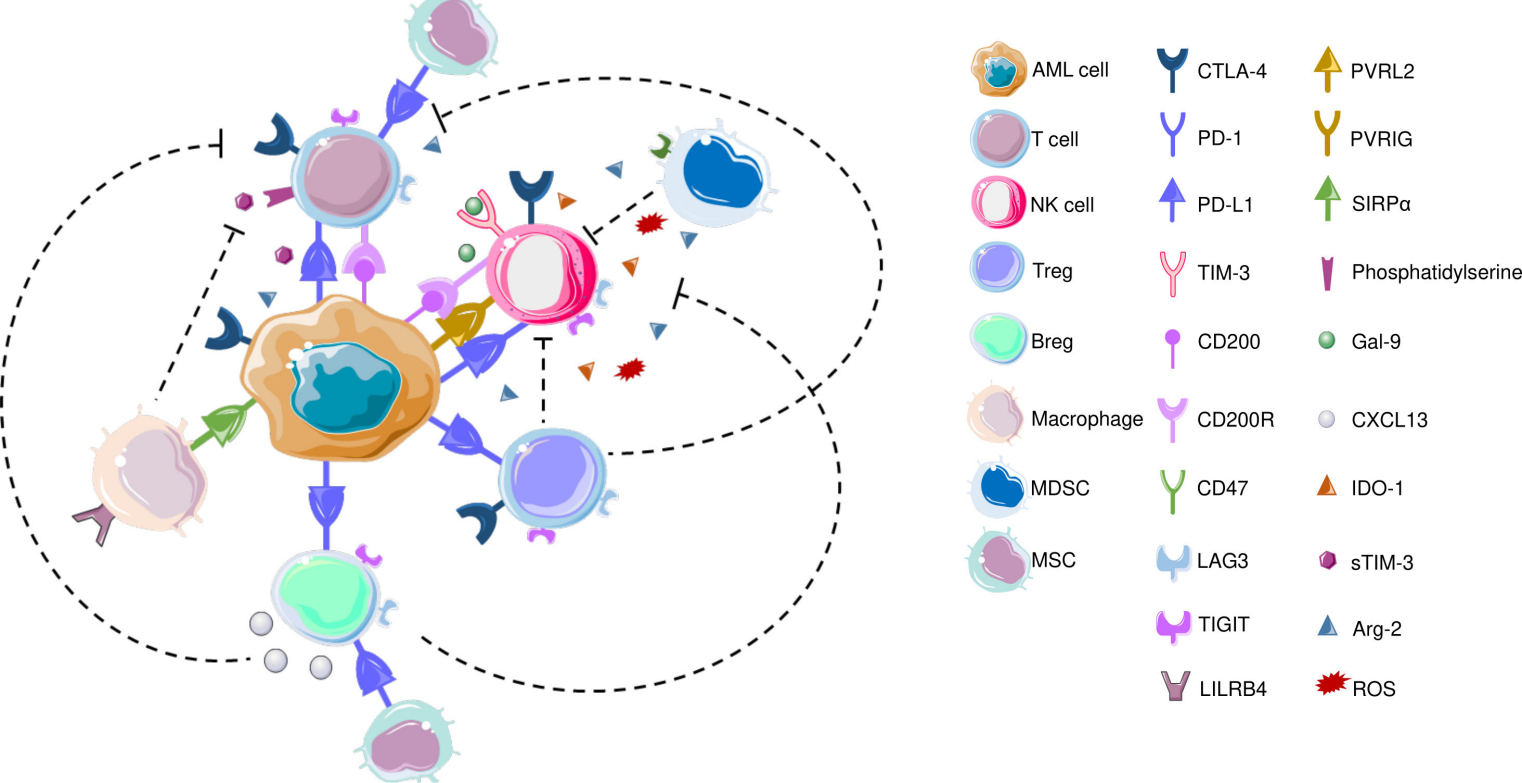
CTLA-4, which is also expressed on T cells and NK cells is the first ICP that is reported to be commonly overexpressed in AML to inhibit T cell responses. In terms of T cells, increased frequency of PD-1+CD4+ T cells as well as PD-1+/CD8+ cells co-expressing TIM3 or LAG3 were reported in AML patients’ bone marrow samples. LSCs also secrete Gal-9 that leads to the elimination Th1 effector cells. LILRB4 is expressed on monocytic leukemic cells and interact with T cells to alter their function. CD200 is also expressed on AML cells that engage in CD200R on T cells and NK cells. Similar to T cells, PD-L1 expression has been detected on Bregs in AML patients. Recently, blocking PD1/PD-L1 axis along with inhibiting CXCL13 has been increased chemotherapeutic efficacy, and CXCL13 has been suggested as a novel ICP; TIGIT is also expressed on BREGs, though both these findings are yet to be confirmed in AML BM samples. TAMs express CD47 that protects phagocytosis of AML LSCs. AML: acute myeloid leukemia; Arg-2: Arginase 2; Breg: B regulatory cell; CTLA-4: Cytotoxic T-lymphocyte antigen-4; CXCL-13: CXC chemokine ligand 13; Gal-9: Galectin-9; IDO-1: Indoleamine 2,3-dioxygenase; LAG3: Lymphocyte-activation gene 3; LILRB4: Leukocyte immunoglobulin like receptor B4; MDSC: myeloid-derived suppressor cell; MSC: Mesenchymal stem cell; NK cell: Natural killer cell; PD-1: Programmed death – 1; PD-L1: Programmed death ligand 1; PVRIG: Poliovirus receptor related immunoglobulin domain containing; PVRL2: Poliovirus receptor-related 2 (Nectin-2); ROS: Reactive oxygen species; SIRPα: Signal regulatory protein α; sTIM-3: soluble TIM-3; TIGIT: T cell immunoreceptor with Ig and ITIM domains; TIM-3: T-cell immunoglobulin and mucin domain 3; Treg: T regulatory cell; VISTA: V-domain Ig suppressor of T cell activation.

AML blasts modulate TME to enable disease progression, provide protection against therapeutic interventions and contribute to recurrence (151). In terms of ICPs, AML blasts can alter the T cell immunological synapses, promote inhibitory soluble factors to hamper T cell responses, and promote activity of MDSCs as well as promoting polarization of tumor associated macrophages (TAMs) to immunomodulatory M2 phenotype (151, 152). The interaction between AML cells and immune cells are visualized in **Figure 2**.

#### 4.2.1 Leukemic cells

PD-1/PD-L1 axis is the most studied IC pathway in AML (153), and PD-L1 expression on AML blasts were reported to be linked with the inflamed tumor microenvironment, highlighting the potential of targeting BMM in disease management (154, 155). In addition, AML cells also secrete soluble ICPs to the microenvironment to create an immunosuppressive milieu as human AML cells including leukemic stem cells have higher TIM-3 and its’ ligand Gal-9 expression levels compared to healthy HSCs. By binding TIM-3 expressed on NK cells, Gal-9 can inhibit granzyme B transfer, and in turn, NK-mediated cell lysis while soluble TIM-3 can suppress IL-2 production by T cells, hampering NK and CTL activation (156).

#### 4.2.2 Endothelial cells

Bone marrow endothelial cells are an important part of the BMM; by secreting growth factors along with certain cytokines and forming physical contact with hematopoietic progenitors, they take part in the regulation of hematopoiesis (157). In cancer, tumor vessels are highly abnormal, and they favor immune suppression (158). T cells can remodel the ECM by downregulating adhesion molecules to prevent infiltration and recruitment of effector immune cells to the cancer milieu; the production of immunosuppressive metabolites, chemokines and cytokines inhibit CTL function while promoting M2 macrophages and MDSCs (159, 160). Thus, normalization of the cancer vasculature would improve immune cell infiltration, promote the immune reactivity, and hamper immune suppressive microenvironment: inhibition of angiogenesis by drugs targeting VEGF-dependent signaling pathways were suggested to improve immunotherapy outcomes (161).

#### 4.2.3 T cells

T cell function holds great importance in IC blockade since they are the main targets of ICIs which are interfered by MDSCs that lead to poor clinical outcome in ICI treatment (162). In AML, certain clinical studies have revealed disruptions in T cell immunity such as increased Treg frequency, reduced T helper cells, increased T cell exhaustion (19). Resident T cells from AML bone marrow samples of AML patients were reported to have altered transcription profiles expressing genes related stemness and myeloid priming (163). Increased frequency of PD-1+CD4+ and ICOS+/CD4+ effector T cells were reported in the BM samples of AML patients (164, 165). In terms of Tregs, their proportion in the BMM was reported to be higher compared to healthy controls, and a higher frequency of PD-1^+^/CD8^+^ cells co-expressing TIM3 or LAG3 was detected, especially in patients who had multiply relapsed AML. Secreted by LSCs, Gal-9 promotes apoptosis of Th1 effector cells and CTLs expressing TIM-3 that eventually leads to T cell exhaustion and immune evasion (166, 167). In TP53-mutated AML patients, leukemia blasts from BMM were more frequently positive for PD-L1 (164). Even after allogeneic HSCT, T cells infiltrating the bone marrow were reported to have early differentiated memory stem (TSCM) and central memory bone marrow-T cell features with multiple IC receptor expressions (168). Another mechanism that inhibit T cell growth is the expression of immunoglobulin-like receptor B4 (LILRB4) which is exclusively expressed on monocytic leukemic cells (M4 and M5 subtypes) that interact with T cells to alter their function *via* releasing arginase-1 to suppress T cell proliferation (169). The immune-suppressive molecule, CD200 is also increased on AML cells to interact with CD200 receptor (CD200R) on T cells to inhibit memory T cell function and increase Treg populations (170).

#### 4.2.4 B cells

Regulatory B cells (Bregs), immunomodulatory B cells that exert immunomodulatory effects mainly *via* secreting various soluble mediators including IL-10 are reported to increase in peripheral blood as well as bone marrow samples in AML patients, highlighting their role in the AML pathogenesis (171). Recently, PD-L1 expression has been reported on Bregs in AML patients and is associated with a worse prognosis (165). According to an *in-vivo* study, CXCL13 has been suggested as a novel IC regulating Breg activity where ablation of CXCL13 increased the efficacy of chemotherapy and PD-1 blockade, though this study did not involve an AML model (172). Other ICPs involved in Bregs’ involve TIGIT, although its’ mechanism of action in AML is yet to be elucidated (173).

#### 4.2.5 Myeloid-derived suppressor cells

Myeloid-derived suppressor cells (MDSCs) are a heterogenous group of CD11b+ CD33+ HLA-DR^lo/neg^ immature myeloid cells that consist of three major groups: monocytic MDSCs (M-MDSCs, CD11b+CD14+HLA-DR^lo^) that resemble monocytes in terms of their phenotypes and morphologies, polymorphonuclear MDSCs (PMN-MDSCs, CD11b+CD15+CD14^neg^) that are similar to neutrophils and early-stage MDSC (eMDSC, CD11b+CD33+CD14^neg^CD15^neg^HLA-DR^neg^) (174–176). All subsets of MDSCs are known to exert immunosuppressive effects, both at a systemic and at the tumor level which led to the investigations questioning their potential for being biomarkers in response to ICI (176). In pathological conditions including cancer, MDSCs expand in response to inflammatory mediators as well as growth factors released, and they undergo expansion to participate in disease development. The presence of circulating M-MDSCs may correlate with response to anti-PD-1 treatments: advanced melanoma patients with lower circulating M-MDSCs levels prior to nivolumab treatment had shown better response to treatment, and Gal-9 expression of M-MDSCs is shown to be associated with TIM-3 expression on lymphocytes which contributes to nivolumab resistance in non-small cell lung carcinoma. In AML, expansion of MDSCs were shown to suppress T-cell proliferation and T-cell responses while MDSC expansion was reported to be Muc-1 mediated c-myc expression dependent, which has shown to be associated with PD-L1 expression in AML cases with TP53 mutations (177). VISTA has been found to be highly expressed on MDSCs, and knockdown of this ICP reduced MDSC-mediated CD8+ T cell inhibition (62). Previously, cytarabine in combination with CXCR4 inhibitor plerixafor and anti-PD-L1 monoclonal antibody have successfully decreased Tregs and MDSCs in the peripheral blood and leukemic cells in the bone marrow (178).

#### 4.2.6 Tumor associated macrophages

Polarization of TAMs to anti-inflammatory M2 phenotype has been well documented in AML, which hampers anti-tumor immunity and promotes cancer progression (179, 180). A study published by Al-Matary et al. revealed that AML increases invasion of TAMs in the BM and spleen in mice as well as leukemic patients, and Growth factor independence-1 is the main regulator of M2 polarization (181). Novel macrophage ICP, CD47 plays important role in various cancers, mainly in myeloid malignancies and it is recognized as an LSC marker in AML (58). CD47 prevents phagocytosis of AML leukemic stem cells by interacting with SIRPα, and inhibition of this pathway replenishes the phagocytosis ability of TAMs to engulf AML LSCs (182). In line with these findings, the anti-CD47 antibody magrolimab was revealed to show promising results when combined with azacitidine in AML and MDS patients (58), and a phase 3 study evaluating the efficacy of magrolimab in combination with venetoclax and azacytidine has been ongoing (183). As magrolimab promotes phagocytosis by interacting Fc gamma receptors on macrophages, the mechanism of action of the monoclonal antibody raised questions in terms of its’ toxicity as CD47 is also expressed on healthy cells (58, 184). However, inhibition of CD47 only promoted phagocytosis if prophagocytic signals are present, which are normally absent on healthy cells (184).

#### 4.2.7 Natural killer cells

Along with T cells, NK cells target AML blasts *via* MHC molecules, leukemia-associated antigens (LAAs), or NK cell activating ligands (185), and ratio of NK cells in the BM samples of the patients has been shown to be correlated with better prognosis (186). However, AML can modulate NK cell activity to eliminate anti-leukemic responses by altering expression of ligands and receptors (187), and studies revealed a correlation between AML blast ligand repertoire and NK receptor expression in patients receiving chemotherapy (188, 189). Recently, NK cells are reported to express PD-1, and inhibiting the PD-1/PD-L1 axis has been shown to activate these cells, suggesting NK activation as another result of ICI administration (190). However, it should be noted that this study did not involve AML patients but murine cancer models, and the functional effects of PD-1 engagement on NK cells was investigated *in-vitro*. Another recent study highlighted the involvement of PVRIG/PVRL2 axis in AML and suggested that PVRIG blockade may be a novel approach to enhance NK cell activity in PVRL2+ AML (57). Besides PD-1 and PVRIG, expression of TIM-3, LAG3, TIGIT, Siglec-7/9, CD200R, CTLA-4, or B7H3 were also reported on NK cells, though in a lesser extent in comparison with T cells (191, 192). It should be noted that none of these studies focus on the activity of NK cells with an emphasis on AML, indicating the requirement of further analyses regarding the NK cell-mediated anti-leukemic mechanisms of ICI in AML.

#### 4.2.8 Mesenchymal stem cells

MSCs influence their microenvironment by interacting with neighboring cells *via* direct contact or secreting various mediators that regulate innate and adaptive immune cells (193). MSCs inhibit the function of T cells, NK cells; suppress dendritic cells’ maturation, and promote Tregs’ proliferation (194). MSCs also support hematopoiesis and promote HSCs’ colonization, and sharing the same microenvironment with HSCs, leukemic stem cells can modulate MSCs immunomodulatory actions: in AML, Nestin+ BM-MSCs were reported to have altered properties that contribute to disease development and chemoresistance (119). Under inflammatory conditions, MSCs are reported to produce PD-L1 and PD-L2 which bind to PD-1 on T cells to inhibit their activation and contribute to immune escape (195). However, our current knowledge regarding ICP expression on MSCs are limited, and further studies on ICI-mediated anti-leukemic effects of MSCs are required.

#### 4.2.9 Adipocytes

Bone marrow adipocytes (BMAs) are thought to be differentiated from Sca1+ CD45− CD31− or LepR+ CD45− CD31− MSCs (196, 197). These small adipocytes secrete high levels of adipokines but have lower CD36 and triglyceride levels compared to white adipose tissue, and they do not share the same progenitors with brown adipose tissue and contribute to inflammation by secreting high levels of proinflammatory cytokines (198, 199). In 2018, Wu et al. demonstrated PD-L1 gene expression in murine adipose tissue and indicated that inducing adipogenesis in mouse cell lines *in vitro* enhanced its’ expression up to 100-fold (200). Recently, Picarda et al. reported that ICP B7-H3 is expressed on both mouse and human adipocyte progenitors and involve in the glycolytic and mitochondrial activity while its’ loss upon adipocytic differentiation results in impaired oxidative metabolism and increased lipid accumulation (201). However, none of these studies involve BMAs; when considering their unique properties, expression levels of ICPs and their involvement in the regulation of hematopoiesis as well as leukemia initiation and progression all require further studies.

## 5 Conclusions and future perspectives

Today it is widely known that the structure and the function of BMM is altered to facilitate AML progression, dissemination and escape immune surveillance (202). Manipulation of the CXCL12/CXCR4 pathway is the key player in AML blasts’ growth, survival, and chemotherapy resistance: CXCR4 expression on AML blasts that is involved in trafficking of malignant LSCs within BM while the migration of healthy stem cells in BM is prohibited (22). Regulation of tumor immune microenvironment stands out as a promising strategy in cancer treatment; in AML, inhibitors of several pathways are currently being investigated, either alone or in combination (203). When considering the therapeutic interventions targeting tumor microenvironment can alter ICP expression in tumor microenvironment, inhibiting ICPs on AML blasts and stem cells may be regarded as a combinatorial treatment strategy. In colorectal cancer, HMA decitabine enhanced the therapeutic efficacy of PD-L1 blockade and in ovarian cancer, dual inhibition of CXCL12-CXCR4 and PD-1-PD-L1 axes alleviated the immunosuppressive tumor microenvironment (204, 205). While these data underline the potential of ICP blockade in AML treatment *via* BMM modulation, it should be noted that our current knowledge regarding ICIs mainly relies on studies with solid tumors, and more data involving larger patient cohorts are needed to determine whether they will be integrated into therapeutic routines in hematological malignancies, and the impact of tumor immune microenvironment on the success of ICIs require more investigation.

## Author contributions

Drafting manuscript: BA, CP, ZD, NND-C and EG. Preparing the figures: BA and NND-C. Editing and revising the manuscript: GY-D. All authors contributed to the article and approved the submitted version.

## References

[1] Arber DA Orazi A Hasserjian R Thiele J Borowitz MJ Le Beau MM. The 2016 revision to the world health organization classification of myeloid neoplasms and acute leukemia. Blood (2016) 127(20):2391–405. doi: 10.1182/blood-2016-03-643544 27069254

[2] Jaffe E Arber D Campo E Harris N Quintanilla-Fend L. Hematopathology. 2nd ed. New York: Elsevier (2016).

[3] Narayanan D Weinberg OK. How I investigate acute myeloid leukemia. Int J Lab Hematol (2020) 42(1):3–15. doi: 10.1111/ijlh.13135 31820579

[4] Jalaeikhoo H Kashfi SMH Azimzadeh P Narimani A Gouhari Moghadam K Rajaienejad M. Acute myeloid leukemia as the main cause of pancytopenia in Iranian population. Iran J Pathol (2017) 12(3):265–71. doi: 10.30699/ijp.2017.25647 PMC5835375 29531552

[5] Lad D Jain A Varma S. Complications and management of coagulation disorders in leukemia patients. Blood Lymphat Cancer (2017) 7:61–72. doi: 10.2147/blctt.S125121 31360085 PMC6467343

[6] Ferraro F Miller CA Christensen KA Helton NM O’Laughlin M Fronick CC. Immunosuppression and outcomes in adult patients with *De novo* acute myeloid leukemia with normal karyotypes. Proc Natl Acad Sci U.S.A. (2021) 118(49):e2116427118. doi: 10.1073/pnas.2116427118 34845035 PMC8673586

[7] Uchiumi H Matsushima T Yamane A Doki N Irisawa H Saitoh T. Prevalence and clinical characteristics of acute myeloid leukemia associated with disseminated intravascular coagulation. Int J Hematol (2007) 86(2):137–42. doi: 10.1532/ijh97.06173 17875527

[8] Stahl M Shallis RM Wei W Montesinos P Lengline E Neukirchen J. Management of hyperleukocytosis and impact of leukapheresis among patients with acute myeloid leukemia (Aml) on short- and long-term clinical outcomes: A Large, retrospective, multicenter, international study. Leukemia (2020) 34(12):3149–60. doi: 10.1038/s41375-020-0783-3 PMC8155811 32132655

[9] Rico-Rodríguez J Villanueva-Ortiz Á Santana-Cabrera L Rodríguez-Pérez H. Pulmonary leukostasis with severe respiratory impairment as a debut of acute myeloid leukemia. Int J Crit Illn Inj Sci (2015) 5(2):125–6. doi: 10.4103/2229-5151.158423 PMC4477392 26157660

[10] Nabhan C Kamat S Karl Kish J. Acute myeloid leukemia in the elderly: What constitutes treatment value? Leuk Lymphoma (2019) 60(5):1164–70. doi: 10.1080/10428194.2018.1520992 30407103

[11] Weinberg OK Hasserjian RP Baraban E Ok CY Geyer JT Philip J. Clinical, immunophenotypic, and genomic findings of acute undifferentiated leukemia and comparison to acute myeloid leukemia with minimal differentiation: A study from the bone marrow pathology group. Mod Pathol (2019) 32(9):1373–85. doi: 10.1038/s41379-019-0263-3 31000771

[12] Franchini M Frattini F Crestani S Bonfanti C. Bleeding complications in patients with hematologic malignancies. Semin Thromb Hemost (2013) 39(1):94–100. doi: 10.1055/s-0032-1331154 23247655

[13] Jongen-Lavrencic M Grob T Hanekamp D Kavelaars FG Al Hinai A Zeilemaker A. Molecular minimal residual disease in acute myeloid leukemia. N Engl J Med (2018) 378(13):1189–99. doi: 10.1056/NEJMoa1716863 29601269

[14] National Comprehensive Cancer Network. Nccn clinical practice guidelines in oncology: Acute myeloid leukemia (2022). Available at: https://www.nccn.org/login?ReturnURL=https://www.nccn.org/professionals/physician_gls/pdf/aml.pdf.

[15] National Comprehensive Cancer Network. Nccn clinical practice guidelines in oncology (Nccn guidelines®): Acut myeloid leukemia nccn evidence blocks (2022). Available at: https://www.nccn.org/professionals/physician_gls/pdf/aml_blocks.pdf.

[16] Ishii H Yano S. New therapeutic strategies for adult acute myeloid leukemia. Cancers (Basel) (2022) 14(11):2806. doi: 10.3390/cancers14112806 35681786 PMC9179253

[17] Ball B Mei M Otoukesh S Stein A. Current and emerging therapies for acute myeloid leukemia. In: Pullarkat V Marcucci G, editors. Biology and treatment of leukemia and bone marrow neoplasms. Cham: Springer International Publishing (2021). p. 57–73. 10.1007/978-3-030-78311-2_4 34626355

[18] Bakst RL Dabaja BS Specht LK Yahalom J. Use of radiation in extramedullary Leukemia/Chloroma: Guidelines from the international lymphoma radiation oncology group. Int J Radiat Oncol Biol Phys (2018) 102(2):314–9. doi: 10.1016/j.ijrobp.2018.05.045 30191865

[19] Hao F Sholy C Wang C Cao M Kang X. The role of T cell immunotherapy in acute myeloid leukemia. Cells (2021) 10(12):3376. doi: 10.3390/cells10123376 34943884 PMC8699747

[20] Döhner H Wei AH Appelbaum FR Craddock C DiNardo CD Dombret H. Diagnosis and management of aml in adults: 2022 recommendations from an international expert panel on behalf of the eln. Blood (2022) 140(12):1345–77. doi: 10.1182/blood.2022016867 35797463

[21] Stubbins RJ Francis A Kuchenbauer F Sanford D. Management of acute myeloid leukemia: A review for general practitioners in oncology. Curr Oncol (2022) 29(9):6245–59. doi: 10.3390/curroncol29090491 PMC9498246 36135060

[22] Hino C Pham B Park D Yang C Nguyen MHK Kaur S. Targeting the tumor microenvironment in acute myeloid leukemia: The future of immunotherapy and natural products. Biomedicines (2022) 10(6):1410. doi: 10.3390/biomedicines10061410 35740430 PMC9219790

[23] Reis M Ogonek J Qesari M Borges NM Nicholson L Preußner L. Recent developments in cellular immunotherapy for hsct-associated complications. Front Immunol (2016) 7:500. doi: 10.3389/fimmu.2016.00500 27895644 PMC5107577

[24] Dessie G Derbew Molla M Shibabaw T Ayelign B. Role of stem-cell transplantation in leukemia treatment. Stem Cells Cloning (2020) 13:67–77. doi: 10.2147/sccaa.S262880 32982314 PMC7493021

[25] Fathi AT. Antibody-based therapy in aml: Antibody–drug conjugates and bispecific agents. Clin Lymphoma Myeloma Leukemia (2021) 21:S112–S3. doi: 10.1016/S2152-2650(21)01231-3

[26] Jabbour E Paul S Kantarjian H. The clinical development of antibody-drug conjugates - lessons from leukaemia. Nat Rev Clin Oncol (2021) 18(7):418–33. doi: 10.1038/s41571-021-00484-2 33758376

[27] Kovtun Y Jones GE Adams S Harvey L Audette CA Wilhelm A. A Cd123-targeting antibody-drug conjugate, Imgn632, designed to eradicate aml while sparing normal bone marrow cells. Blood Adv (2018) 2(8):848–58. doi: 10.1182/bloodadvances.2018017517 PMC5916008 29661755

[28] Imgn632 as monotherapy or with venetoclax and/or azacitidine for patients with Cd123-positive acute myeloid leukemia . Available at: https://clinicaltrials.gov/ct2/show/NCT04086264.

[29] Taghiloo S Asgarian-Omran H. Immune evasion mechanisms in acute myeloid leukemia: A focus on immune checkpoint pathways. Crit Rev Oncol Hematol (2021) 157:103164. doi: 10.1016/j.critrevonc.2020.103164 33271388

[30] Aru B Soltani M Pehlivanoglu C Gurlu E Ganjalikhani-Hakemi M Yanikkaya Demirel G. Comparison of laboratory methods for the clinical follow up of checkpoint blockade therapies in leukemia: Current status and challenges ahead. Front Oncol (2022) 12:789728. doi: 10.3389/fonc.2022.789728 35155232 PMC8829140

[31] Shiravand Y Khodadadi F Kashani SMA Hosseini-Fard SR Hosseini S Sadeghirad H. Immune checkpoint inhibitors in cancer therapy. Curr Oncol (2022) 29(5):3044–60. doi: 10.3390/curroncol29050247 PMC9139602 35621637

[32] Costa B Vale N. Dostarlimab: A review. Biomolecules (2022) 12(8):1031. doi: 10.3390/biom12081031 35892341 PMC9331682

[33] Pistillo MP Tazzari PL Palmisano GL Pierri I Bolognesi A Ferlito F. Ctla-4 is not restricted to the lymphoid cell lineage and can function as a target molecule for apoptosis induction of leukemic cells. Blood (2003) 101(1):202–9. doi: 10.1182/blood-2002-06-1668 12393538

[34] Zhong RK Loken M Lane TA Ball ED. Ctla-4 blockade by a human mab enhances the capacity of aml-derived dc to induce T-cell responses against aml cells in an autologous culture system. Cytotherapy (2006) 8(1):3–12. doi: 10.1080/14653240500499507 16627340

[35] Saudemont A Quesnel B. In a model of tumor dormancy, long-term persistent leukemic cells have increased B7-H1 and B7.1 expression and resist ctl-mediated lysis. Blood (2004) 104(7):2124–33. doi: 10.1182/blood-2004-01-0064 15191948

[36] Wei SC Anang NAS Sharma R Andrews MC Reuben A Levine JH. Combination anti-Ctla-4 plus anti-Pd-1 checkpoint blockade utilizes cellular mechanisms partially distinct from monotherapies. Proc Natl Acad Sci U.S.A. (2019) 116(45):22699–709. doi: 10.1073/pnas.1821218116 PMC6842624 31636208

[37] Zhang L Gajewski TF Kline J. Pd-1/Pd-L1 interactions inhibit antitumor immune responses in a murine acute myeloid leukemia model. Blood (2009) 114(8):1545–52. doi: 10.1182/blood-2009-03-206672 PMC2731636 19417208

[38] Curran E Chen X Corrales L Kline DE Dubensky TW Jr. Duttagupta P. Sting pathway activation stimulates potent immunity against acute myeloid leukemia. Cell Rep (2016) 15(11):2357–66. doi: 10.1016/j.celrep.2016.05.023 PMC5116809 27264175

[39] Gajewski TF. Failure at the effector phase: Immune barriers at the level of the melanoma tumor microenvironment. Clin Cancer Res (2007) 13(18 Pt 1):5256–61. doi: 10.1158/1078-0432.Ccr-07-0892 17875753

[40] Fuertes MB Kacha AK Kline J Woo SR Kranz DM Murphy KM. Host type I ifn signals are required for antitumor Cd8+ T cell responses through Cd8α+ dendritic cells. J Exp Med (2011) 208(10):2005–16. doi: 10.1084/jem.20101159 PMC3182064 21930765

[41] Chen H Sun H You F Sun W Zhou X Chen L. Activation of Stat6 by sting is critical for antiviral innate immunity. Cell (2011) 147(2):436–46. doi: 10.1016/j.cell.2011.09.022 22000020

[42] Fu J Kanne DB Leong M Glickman LH McWhirter SM Lemmens E. Sting agonist formulated cancer vaccines can cure established tumors resistant to pd-1 blockade. Sci Transl Med (2015) 7(283):283ra52. doi: 10.1126/scitranslmed.aaa4306 PMC4504692 25877890

[43] Zhang L Chen X Liu X Kline DE Teague RM Gajewski TF. Cd40 ligation reverses T cell tolerance in acute myeloid leukemia. J Clin Invest (2013) 123(5):1999–2010. doi: 10.1172/jci63980 23619361 PMC3635717

[44] Przespolewski A Portwood S Den Haese J Lewis D Wang ES. Targeting innate and adaptive immune responses for the treatment of acute myeloid leukemia. Blood (2016) 128(22):2833–. doi: 10.1182/blood.V128.22.2833.2833

[45] Przespolewski AC Portwood S Wang ES. Targeting acute myeloid leukemia through multimodal immunotherapeutic approaches. Leuk Lymphoma (2022) 63(4):918–27. doi: 10.1080/10428194.2021.1992614 PMC10691526 34818963

[46] Adam M Yu J Plant R Shelton C Schmidt H Yang J. Sting agonist Gsk3745417 induces apoptosis, antiproliferation, and cell death in a panel of human aml cell lines and patient samples. Blood (2022) 140(Supplement 1):11829–. doi: 10.1182/blood-2022-167652

[47] Meric-Bernstam F Sweis RF Hodi FS Messersmith WA Andtbacka RHI Ingham M. Phase I dose-escalation trial of Miw815 (Adu-S100), an intratumoral sting agonist, in patients with Advanced/Metastatic solid tumors or lymphomas. Clin Cancer Res (2022) 28(4):677–88. doi: 10.1158/1078-0432.Ccr-21-1963 34716197

[48] Song C Liu D Liu S Li D Horecny I Zhang X. Shr1032, a novel sting agonist, stimulates anti-tumor immunity and directly induces aml apoptosis. Sci Rep (2022) 12(1):8579. doi: 10.1038/s41598-022-12449-1 35595822 PMC9122897

[49] Study of ulevostinag (Mk-1454) alone or in combination with pembrolizumab (Mk-3475) in participants with Advanced/Metastatic solid tumors or lymphomas (Mk-1454-001) (2022). Available at: https://clinicaltrials.gov/ct2/show/NCT03010176.

[50] Phase 1 first time in human (Ftih), open label study of Gsk3745417 administered to participants with advanced solid tumors (2022). Available at: https://clinicaltrials.gov/ct2/show/NCT03843359.

[51] An investigational immunotherapy study of bms-986301 alone or in combination with nivolumab, and ipilimumab in participants with advanced solid cancers (2022). Available at: https://clinicaltrials.gov/ct2/show/NCT03956680.

[52] Motedayen Aval L Pease JE Sharma R Pinato DJ. Challenges and opportunities in the clinical development of sting agonists for cancer immunotherapy. J Clin Med (2020) 9(10):3323. doi: 10.3390/jcm9103323 33081170 PMC7602874

[53] Gonçalves Silva I Rüegg L Gibbs BF Bardelli M Fruehwirth A Varani L. The immune receptor Tim-3 acts as a trafficker in a Tim-3/Galectin-9 autocrine loop in human myeloid leukemia cells. Oncoimmunology (2016) 5(7):e1195535. doi: 10.1080/2162402x.2016.1195535 27622049 PMC5006895

[54] Taghiloo S Norozi S Asgarian-Omran H. The effects of Pi3k/Akt/Mtor signaling pathway inhibitors on the expression of immune checkpoint ligands in acute myeloid leukemia cell line. Iran J Allergy Asthma Immunol (2022) 21(2):178–88. doi: 10.18502/ijaai.v21i2.9225 35490271

[55] Xu L Liu L Yao D Zeng X Zhang Y Lai J. Pd-1 and tigit are highly Co-expressed on Cd8(+) T cells in aml patient bone marrow. Front Oncol (2021) 11:686156. doi: 10.3389/fonc.2021.686156 34490086 PMC8416522

[56] Brauneck F Seubert E Wellbrock J Schulze Zur Wiesch J Duan Y Magnus T. Combined blockade of tigit and Cd39 or A2ar enhances nk-92 cell-mediated cytotoxicity in aml. Int J Mol Sci (2021) 22(23):12919. doi: 10.3390/ijms222312919 34884723 PMC8657570

[57] Li J Whelan S Kotturi MF Meyran D D’Souza C Hansen K. Pvrig is a novel natural killer cell immune checkpoint receptor in acute myeloid leukemia. Haematologica (2021) 106(12):3115–24. doi: 10.3324/haematol.2020.258574 PMC8634199 33147937

[58] Chao MP Takimoto CH Feng DD McKenna K Gip P Liu J. Therapeutic targeting of the macrophage immune checkpoint Cd47 in myeloid malignancies. Front Oncol (2019) 9:1380. doi: 10.3389/fonc.2019.01380 32038992 PMC6990910

[59] Tonks A Hills R White P Rosie B Mills KI Burnett AK. Cd200 as a prognostic factor in acute myeloid leukaemia. Leukemia (2007) 21(3):566–8. doi: 10.1038/sj.leu.2404559 17252007

[60] Zahran AM Mohammed Saleh MF Sayed MM Rayan A Ali AM Hetta HF. Up-regulation of regulatory T cells, Cd200 and Tim3 expression in cytogenetically normal acute myeloid leukemia. Cancer biomark (2018) 22(3):587–95. doi: 10.3233/cbm-181368 PMC13078467 29843224

[61] Kim TK Han X Wang J Sanmamed M Zhang T Halene S. Pd-1h (Vista) induces immune evasion in acute myeloid leukemia. Blood (2017) 130(Supplement 1):2658–. doi: 10.1182/blood.V130.Suppl_1.2658.2658

[62] Wang L Jia B Claxton DF Ehmann WC Rybka WB Mineishi S. Vista is highly expressed on mdscs and mediates an inhibition of T cell response in patients with aml. Oncoimmunology (2018) 7(9):e1469594. doi: 10.1080/2162402x.2018.1469594 30228937 PMC6140587

[63] Radwan SM Elleboudy NS Nabih NA Kamal AM. The immune checkpoints cytotoxic T lymphocyte antigen-4 and lymphocyte activation gene-3 expression is up-regulated in acute myeloid leukemia. Hla (2020) 96(1):3–12. doi: 10.1111/tan.13872 32189430

[64] Herrmann M Krupka C Deiser K Brauchle B Marcinek A Ogrinc Wagner A. Bifunctional pd-1 × Acd3 × Acd33 fusion protein reverses adaptive immune escape in acute myeloid leukemia. Blood (2018) 132(23):2484–94. doi: 10.1182/blood-2018-05-849802 30275109

[65] Assi R Kantarjian H Ravandi F Daver N. Immune therapies in acute myeloid leukemia: A focus on monoclonal antibodies and immune checkpoint inhibitors. Curr Opin Hematol (2018) 25(2):136–45. doi: 10.1097/moh.0000000000000401 29206680

[66] Daver N Alotaibi AS Bücklein V Subklewe M. T-Cell-Based immunotherapy of acute myeloid leukemia: Current concepts and future developments. Leukemia (2021) 35(7):1843–63. doi: 10.1038/s41375-021-01253-x PMC8257483 33953290

[67] Yang H Bueso-Ramos C DiNardo C Estecio MR Davanlou M Geng QR. Expression of pd-L1, pd-L2, pd-1 and Ctla4 in myelodysplastic syndromes is enhanced by treatment with hypomethylating agents. Leukemia (2014) 28(6):1280–8. doi: 10.1038/leu.2013.355 PMC4032802 24270737

[68] Ørskov AD Treppendahl MB Skovbo A Holm MS Friis LS Hokland M. Hypomethylation and up-regulation of pd-1 in T cells by azacytidine in Mds/Aml patients: A rationale for combined targeting of pd-1 and DNA methylation. Oncotarget (2015) 6(11):9612–26. doi: 10.18632/oncotarget.3324 PMC4496243 25823822

[69] Daver N Boddu P Garcia-Manero G Yadav SS Sharma P Allison J. Hypomethylating agents in combination with immune checkpoint inhibitors in acute myeloid leukemia and myelodysplastic syndromes. Leukemia (2018) 32(5):1094–105. doi: 10.1038/s41375-018-0070-8 PMC6916728 29487386

[70] Daver N Garcia-Manero G Basu S Boddu PC Alfayez M Cortes JE. Efficacy, safety, and biomarkers of response to azacitidine and nivolumab in Relapsed/Refractory acute myeloid leukemia: A nonrandomized, open-label, phase ii study. Cancer Discovery (2019) 9(3):370–83. doi: 10.1158/2159-8290.Cd-18-0774 PMC6397669 30409776

[71] Daver NG Garcia-Manero G Konopleva MY Alfayez M Pemmaraju N Kadia TM. Azacitidine (Aza) with nivolumab (Nivo), and aza with nivo + ipilimumab (Ipi) in Relapsed/Refractory acute myeloid leukemia: A non-randomized, prospective, phase 2 study. Blood (2019) 134(Supplement_1):830–. doi: 10.1182/blood-2019-131494

[72] Gojo I Stuart RK Webster J Blackford A Varela JC Morrow J. Multi-center phase 2 study of pembroluzimab (Pembro) and azacitidine (Aza) in patients with Relapsed/Refractory acute myeloid leukemia (Aml) and in newly diagnosed (≥65 years) aml patients. Blood (2019) 134(Supplement_1):832–. doi: 10.1182/blood-2019-127345

[73] Daver N Basu S Garcia-Manero G Cortes JE Ravandi F Jabbour EJ. Phase Ib/Ii study of nivolumab in combination with azacytidine (Aza) in patients (Pts) with relapsed acute myeloid leukemia (Aml). Blood (2016) 128(22):763–. doi: 10.1182/blood.V128.22.763.763

[74] Zeidan AM Cavenagh J Voso MT Taussig D Tormo M Boss I. Efficacy and safety of azacitidine (Aza) in combination with the anti-Pd-L1 durvalumab (Durva) for the front-line treatment of older patients (Pts) with acute myeloid leukemia (Aml) who are unfit for intensive chemotherapy (Ic) and pts with higher-risk myelodysplastic syndromes (Hr-mds): Results from a Large, international, randomized phase 2 study. Blood (2019) 134(Supplement_1):829–. doi: 10.1182/blood-2019-122896

[75] Duan J Cui L Zhao X Bai H Cai S Wang G. Use of immunotherapy with programmed cell death 1 vs programmed cell death ligand 1 inhibitors in patients with cancer: A systematic review and meta-analysis. JAMA Oncol (2020) 6(3):375–84. doi: 10.1001/jamaoncol.2019.5367 PMC6990765 31876895

[76] Herbrich S Cavazos A Cheung CMC Alexander-Williams L Short NJ Matthews J. Single-cell mass cytometry identifies mechanisms of resistance to immunotherapy in aml. Blood (2019) 134(Supplement_1):1428–. doi: 10.1182/blood-2019-128601

[77] Berger R Rotem-Yehudar R Slama G Landes S Kneller A Leiba M. Phase I safety and pharmacokinetic study of ct-011, a humanized antibody interacting with pd-1, in patients with advanced hematologic malignancies. Clin Cancer Res (2008) 14(10):3044–51. doi: 10.1158/1078-0432.Ccr-07-4079 18483370

[78] Boddu P Kantarjian H Garcia-Manero G Allison J Sharma P Daver N. The emerging role of immune checkpoint based approaches in aml and mds. Leuk Lymphoma (2018) 59(4):790–802. doi: 10.1080/10428194.2017.1344905 28679300 PMC5872841

[79] Blockade of pd-1 in conjunction with the dendritic Cell/Aml vaccine following chemotherapy induced remission (2022). Available at: https://clinicaltrials.gov/ct2/show/NCT01096602.

[80] Davids MS Kim HT Bachireddy P Costello C Liguori R Savell A. Ipilimumab for patients with relapse after allogeneic transplantation. N Engl J Med (2016) 375(2):143–53. doi: 10.1056/NEJMoa1601202 PMC5149459 27410923

[81] Zeidner JF Vincent BG Ivanova A Moore D McKinnon KP Wilkinson AD. Phase ii trial of pembrolizumab after high-dose cytarabine in Relapsed/Refractory acute myeloid leukemia. Blood Cancer Discovery (2021) 2(6):616–29. doi: 10.1158/2643-3230.Bcd-21-0070 PMC8580622 34778801

[82] Tschernia NP Kumar V Moore DT Vincent BG Coombs CC Van Deventer H. Safety and efficacy of pembrolizumab prior to allogeneic stem cell transplantation for acute myelogenous leukemia. Transplant Cell Ther (2021) 27(12):1021.e1–.e5. doi: 10.1016/j.jtct.2021.08.022 34474164

[83] Brunner AM Esteve J Porkka K Knapper S Traer E Scholl S. Efficacy and safety of sabatolimab (Mbg453) in combination with hypomethylating agents (Hmas) in patients (Pts) with very High/High-risk myelodysplastic syndrome (Vhr/Hr-mds) and acute myeloid leukemia (Aml): Final analysis from a phase ib study. Blood (2021) 138(Supplement 1):244–. doi: 10.1182/blood-2021-146039

[84] Kadia TM Cortes JE Ghorab A Ravandi F Jabbour E Daver NG. Nivolumab (Nivo) maintenance (Maint) in high-risk (Hr) acute myeloid leukemia (Aml) patients. J Clin Oncol (2018) 36(15_suppl):7014–. doi: 10.1200/JCO.2018.36.15_suppl.7014

[85] Bewersdorf JP Stahl M Zeidan AM. Immune checkpoint-based therapy in myeloid malignancies: A promise yet to be fulfilled. Expert Rev Anticancer Ther (2019) 19(5):393–404. doi: 10.1080/14737140.2019.1589374 30887841 PMC6527485

[86] Silva M Martins D Mendes F. The role of immune checkpoint blockade in acute myeloid leukemia. Onco (2022) 2(3):164–80. doi: 10.3390/onco2030011

[87] Thummalapalli R Knaus HA Gojo I Zeidner JF. Immune checkpoint inhibitors in aml-a new frontier. Curr Cancer Drug Targets (2020) 20(7):545–57. doi: 10.2174/1568009620666200421081455 32316893

[88] Fröbel J Landspersky T Percin G Schreck C Rahmig S Ori A. The hematopoietic bone marrow niche ecosystem. Front Cell Dev Biol (2021) 9:705410. doi: 10.3389/fcell.2021.705410 34368155 PMC8339972

[89] Dammacco F Leone P Silvestris F Racanelli V Vacca A. Chapter 9 - cancer stem cells in multiple myeloma and the development of novel therapeutic strategies. In: Dammacco F Silvestris F, editors. Oncogenomics. (USA: Academic Press) (2019). p. 121–37.

[90] Sendker S Waack K Reinhardt D. Far from health: The bone marrow microenvironment in aml, a leukemia supportive shelter. Children (Basel) (2021) 8(5):371. doi: 10.3390/children8050371 34066861 PMC8150304

[91] Lo Celso C Fleming HE Wu JW Zhao CX Miake-Lye S Fujisaki J. Live-animal tracking of individual haematopoietic Stem/Progenitor cells in their niche. Nature (2009) 457(7225):92–6. doi: 10.1038/nature07434 PMC2820276 19052546

[92] Reagan MR Rosen CJ. Navigating the bone marrow niche: Translational insights and cancer-driven dysfunction. Nat Rev Rheumatol (2016) 12(3):154–68. doi: 10.1038/nrrheum.2015.160 PMC4947935 26607387

[93] Ehninger A Trumpp A. The bone marrow stem cell niche grows up: Mesenchymal stem cells and macrophages move in. J Exp Med (2011) 208(3):421–8. doi: 10.1084/jem.20110132 PMC3058583 21402747

[94] Winkler IG Sims NA Pettit AR Barbier V Nowlan B Helwani F. Bone marrow macrophages maintain hematopoietic stem cell (Hsc) niches and their depletion mobilizes hscs. Blood (2010) 116(23):4815–28. doi: 10.1182/blood-2009-11-253534 20713966

[95] Canalis E. Notch signaling in skeletal diseases. In: Zaidi M, editor. Encyclopedia of bone biology. Oxford: Academic Press (2020). p. 130–40.

[96] Peci F Dekker L Pagliaro A van Boxtel R Nierkens S Belderbos M. The cellular composition and function of the bone marrow niche after allogeneic hematopoietic cell transplantation. Bone Marrow Transplant (2022) 57(9):1357–64. doi: 10.1038/s41409-022-01728-0 PMC9187885 35690693

[97] Itkin T Gur-Cohen S Spencer JA Schajnovitz A Ramasamy SK Kusumbe AP. Distinct bone marrow blood vessels differentially regulate haematopoiesis. Nature (2016) 532(7599):323–8. doi: 10.1038/nature17624 PMC6450701 27074509

[98] Winkler IG Barbier V Nowlan B Jacobsen RN Forristal CE Patton JT. Vascular niche e-selectin regulates hematopoietic stem cell dormancy, self renewal and chemoresistance. Nat Med (2012) 18(11):1651–7. doi: 10.1038/nm.2969 23086476

[99] Kenswil KJG Jaramillo AC Ping Z Chen S Hoogenboezem RM Mylona MA. Characterization of endothelial cells associated with hematopoietic niche formation in humans identifies il-33 as an anabolic factor. Cell Rep (2018) 22(3):666–78. doi: 10.1016/j.celrep.2017.12.070 29346765

[100] Ding L Saunders TL Enikolopov G Morrison SJ. Endothelial and perivascular cells maintain haematopoietic stem cells. Nature (2012) 481(7382):457–62. doi: 10.1038/nature10783 PMC3270376 22281595

[101] Renders S Svendsen AF Panten J Rama N Maryanovich M Sommerkamp P. Niche derived netrin-1 regulates hematopoietic stem cell dormancy *Via* its receptor neogenin-1. Nat Commun (2021) 12(1):608. doi: 10.1038/s41467-020-20801-0 33504783 PMC7840807

[102] Bruns I Lucas D Pinho S Ahmed J Lambert MP Kunisaki Y. Megakaryocytes regulate hematopoietic stem cell quiescence through Cxcl4 secretion. Nat Med (2014) 20(11):1315–20. doi: 10.1038/nm.3707 PMC4258871 25326802

[103] Nakamura-Ishizu A Takubo K Fujioka M Suda T. Megakaryocytes are essential for hsc quiescence through the production of thrombopoietin. Biochem Biophys Res Commun (2014) 454(2):353–7. doi: 10.1016/j.bbrc.2014.10.095 25451253

[104] Zhao M Perry JM Marshall H Venkatraman A Qian P He XC. Megakaryocytes maintain homeostatic quiescence and promote post-injury regeneration of hematopoietic stem cells. Nat Med (2014) 20(11):1321–6. doi: 10.1038/nm.3706 25326798

[105] Rankin EB Wu C Khatri R Wilson TL Andersen R Araldi E. The hif signaling pathway in osteoblasts directly modulates erythropoiesis through the production of epo. Cell (2012) 149(1):63–74. doi: 10.1016/j.cell.2012.01.051 22464323 PMC3408231

[106] Stier S Ko Y Forkert R Lutz C Neuhaus T Grünewald E. Osteopontin is a hematopoietic stem cell niche component that negatively regulates stem cell pool size. J Exp Med (2005) 201(11):1781–91. doi: 10.1084/jem.20041992 PMC2213260 15928197

[107] Taichman RS Emerson SG. Human osteoblasts support hematopoiesis through the production of granulocyte colony-stimulating factor. J Exp Med (1994) 179(5):1677–82. doi: 10.1084/jem.179.5.1677 PMC2191506 7513014

[108] Jung Y Wang J Song J Shiozawa Y Wang J Havens A. Annexin ii expressed by osteoblasts and endothelial cells regulates stem cell adhesion, homing, and engraftment following transplantation. Blood (2007) 110(1):82–90. doi: 10.1182/blood-2006-05-021352 17360942 PMC1896130

[109] Nakamura Y Arai F Iwasaki H Hosokawa K Kobayashi I Gomei Y. Isolation and characterization of endosteal niche cell populations that regulate hematopoietic stem cells. Blood (2010) 116(9):1422–32. doi: 10.1182/blood-2009-08-239194 20472830

[110] Arai F Hirao A Ohmura M Sato H Matsuoka S Takubo K. Tie2/Angiopoietin-1 signaling regulates hematopoietic stem cell quiescence in the bone marrow niche. Cell (2004) 118(2):149–61. doi: 10.1016/j.cell.2004.07.004 15260986

[111] Yoshihara H Arai F Hosokawa K Hagiwara T Takubo K Nakamura Y. Thrombopoietin/Mpl signaling regulates hematopoietic stem cell quiescence and interaction with the osteoblastic niche. Cell Stem Cell (2007) 1(6):685–97. doi: 10.1016/j.stem.2007.10.020 18371409

[112] Jung Y Wang J Schneider A Sun YX Koh-Paige AJ Osman NI. Regulation of sdf-1 (Cxcl12) production by osteoblasts; a possible mechanism for stem cell homing. Bone (2006) 38(4):497–508. doi: 10.1016/j.bone.2005.10.003 16337237

[113] Asada N Katayama Y Sato M Minagawa K Wakahashi K Kawano H. Matrix-embedded osteocytes regulate mobilization of hematopoietic Stem/Progenitor cells. Cell Stem Cell (2013) 12(6):737–47. doi: 10.1016/j.stem.2013.05.001 23746979

[114] Yamazaki S Ema H Karlsson G Yamaguchi T Miyoshi H Shioda S. Nonmyelinating schwann cells maintain hematopoietic stem cell hibernation in the bone marrow niche. Cell (2011) 147(5):1146–58. doi: 10.1016/j.cell.2011.09.053 22118468

[115] Fallati A Di Marzo N D’Amico G Dander E. Mesenchymal stromal cells (Mscs): An ally of b-cell acute lymphoblastic leukemia (B-all) cells in disease maintenance and progression within the bone marrow hematopoietic niche. Cancers (Basel) (2022) 14(14):3303. doi: 10.3390/cancers14143303 35884364 PMC9323332

[116] DiMascio L Voermans C Uqoezwa M Duncan A Lu D Wu J. Identification of adiponectin as a novel hemopoietic stem cell growth factor. J Immunol (2007) 178(6):3511–20. doi: 10.4049/jimmunol.178.6.3511 17339446

[117] Hoffman R Marcellino BK. Bone marrow microenvironment in health and disease. In: Zaidi M, editor. Encyclopedia of bone biology, vol. . p . Oxford: Academic Press (2020). p. 1–11.

[118] Yao Y Li F Huang J Jin J Wang H. Leukemia stem cell-bone marrow microenvironment interplay in acute myeloid leukemia development. Exp Hematol Oncol (2021) 10(1):39. doi: 10.1186/s40164-021-00233-2 34246314 PMC8272391

[119] Forte D García-Fernández M Sánchez-Aguilera A Stavropoulou V Fielding C Martín-Pérez D. Bone marrow mesenchymal stem cells support acute myeloid leukemia bioenergetics and enhance antioxidant defense and escape from chemotherapy. Cell Metab (2020) 32(5):829–43.e9. doi: 10.1016/j.cmet.2020.09.001 32966766 PMC7658808

[120] Bendall LJ Kortlepel K Gottlieb DJ. Human acute myeloid leukemia cells bind to bone marrow stroma *Via* a combination of beta-1 and beta-2 integrin mechanisms. Blood (1993) 82(10):3125–32. doi: 10.1182/blood.V82.10.3125.3125 7693037

[121] Bendall LJ Makrynikola V Hutchinson A Bianchi AC Bradstock KF Gottlieb DJ. Stem cell factor enhances the adhesion of aml cells to fibronectin and augments fibronectin-mediated anti-apoptotic and proliferative signals. Leukemia (1998) 12(9):1375–82. doi: 10.1038/sj.leu.2401136 9737685

[122] Ries C Loher F Zang C Ismair MG Petrides PE. Matrix metalloproteinase production by bone marrow mononuclear cells from normal individuals and patients with acute and chronic myeloid leukemia or myelodysplastic syndromes. Clin Cancer Res (1999) 5(5):1115–24. 10353746

[123] Lin LI Lin DT Chang CJ Lee CY Tang JL Tien HF. Marrow matrix metalloproteinases (Mmps) and tissue inhibitors of mmp in acute leukaemia: Potential role of mmp-9 as a surrogate marker to monitor leukaemic status in patients with acute myelogenous leukaemia. Br J Haematol (2002) 117(4):835–41. doi: 10.1046/j.1365-2141.2002.03510.x 12060118

[124] Kuek V Hughes AM Kotecha RS Cheung LC. Therapeutic targeting of the leukaemia microenvironment. Int J Mol Sci (2021) 22(13):6888. doi: 10.3390/ijms22136888 34206957 PMC8267786

[125] Noh H Hu J Wang X Xia X Satelli A Li S. Immune checkpoint regulator pd-L1 expression on tumor cells by contacting Cd11b positive bone marrow derived stromal cells. Cell Commun Signal (2015) 13:14. doi: 10.1186/s12964-015-0093-y 25889536 PMC4353689

[126] Dias S Shmelkov SV Lam G Rafii S. Vegf(165) promotes survival of leukemic cells by Hsp90-mediated induction of bcl-2 expression and apoptosis inhibition. Blood (2002) 99(7):2532–40. doi: 10.1182/blood.v99.7.2532 11895790

[127] Lu W Weng W Zhu Q Zhai Y Wan Y Liu H. Small bone marrow adipocytes predict poor prognosis in acute myeloid leukemia. Haematologica (2018) 103(1):e21–e4. doi: 10.3324/haematol.2017.173492 PMC5777209 29051282

[128] Hanoun M Zhang D Mizoguchi T Pinho S Pierce H Kunisaki Y. Acute myelogenous leukemia-induced sympathetic neuropathy promotes malignancy in an altered hematopoietic stem cell niche. Cell Stem Cell (2014) 15(3):365–75. doi: 10.1016/j.stem.2014.06.020 PMC4156919 25017722

[129] Raaijmakers MH Mukherjee S Guo S Zhang S Kobayashi T Schoonmaker JA. Bone progenitor dysfunction induces myelodysplasia and secondary leukaemia. Nature (2010) 464(7290):852–7. doi: 10.1038/nature08851 PMC3422863 20305640

[130] Kode A Manavalan JS Mosialou I Bhagat G Rathinam CV Luo N. Leukaemogenesis induced by an activating B-catenin mutation in osteoblasts. Nature (2014) 506(7487):240–4. doi: 10.1038/nature12883 PMC4116754 24429522

[131] Krause DS Fulzele K Catic A Sun CC Dombkowski D Hurley MP. Differential regulation of myeloid leukemias by the bone marrow microenvironment. Nat Med (2013) 19(11):1513–7. doi: 10.1038/nm.3364 PMC3827980 24162813

[132] Frisch BJ Ashton JM Xing L Becker MW Jordan CT Calvi LM. Functional inhibition of osteoblastic cells in an in vivo mouse model of myeloid leukemia. Blood (2012) 119(2):540–50. doi: 10.1182/blood-2011-04-348151 PMC3384480 21957195

[133] Bewersdorf JP Zeidan AM. Myeloid-derived suppressor cells: A grey eminence in the aml tumor microenvironment? Expert Rev Anticancer Ther (2022) 22(3):239–41. doi: 10.1080/14737140.2022.2030227 35034557

[134] Zeng Z Xi Shi Y Samudio IJ Wang R-Y Ling X Frolova O. Targeting the leukemia microenvironment by Cxcr4 inhibition overcomes resistance to kinase inhibitors and chemotherapy in aml. Blood (2009) 113(24):6215–24. doi: 10.1182/blood-2008-05-158311 PMC2699240 18955566

[135] Abraham M Klein S Bulvik B Wald H Weiss ID Olam D. The Cxcr4 inhibitor bl-8040 induces the apoptosis of aml blasts by downregulating erk, bcl-2, mcl-1 and cyclin-D1 *Via* altered mir-15a/16-1 expression. Leukemia (2017) 31(11):2336–46. doi: 10.1038/leu.2017.82 28280274

[136] Cho B-S Zeng Z Mu H Wang Z Konoplev S McQueen T. Antileukemia activity of the novel peptidic Cxcr4 antagonist Ly2510924 as monotherapy and in combination with chemotherapy. Blood (2015) 126(2):222–32. doi: 10.1182/blood-2015-02-628677 PMC4497963 26031918

[137] Chen Y Zeng Z Shi Y Jacamo R Ludin C Dembowsky K. Targeting Cxcr4, Sdf1 and beta-adrenergic receptors in the aml microenvironment by novel antagonist Pol6326, G-csf and isoproterenol. Blood (2010) 116(21):2179–. doi: 10.1182/blood.V116.21.2179.2179

[138] Kovacsovics TJ Mims A Salama ME Pantin J Rao N Kosak KM. Combination of the low anticoagulant heparin cx-01 with chemotherapy for the treatment of acute myeloid leukemia. Blood Adv (2018) 2(4):381–9. doi: 10.1182/bloodadvances.2017013391 PMC5858478 29467192

[139] Kuhne MR Mulvey T Belanger B Chen S Pan C Chong C. Bms-936564/Mdx-1338: A fully human anti-Cxcr4 antibody induces apoptosis in vitro and shows antitumor activity in vivo in hematologic malignancies. Clin Cancer Res (2013) 19(2):357–66. doi: 10.1158/1078-0432.Ccr-12-2333 23213054

[140] Chien S Beyerle LE Wood BL Estey EH Appelbaum FR Cardarelli PM. Mobilization of blasts and leukemia stem cells by anti-Cxcr4 antibody bms-936564 (Mdx 1338) in patients with Relapsed/Refractory acute myeloid leukemia. Blood (2013) 122(21):3882–. doi: 10.1182/blood.V122.21.3882.3882

[141] Becker PS Foran JM Altman JK Yacoub A Castro JE Sabbatini P. Targeting the Cxcr4 pathway: Safety, tolerability and clinical activity of ulocuplumab (Bms-936564), an anti-Cxcr4 antibody, in Relapsed/Refractory acute myeloid leukemia. Blood (2014) 124(21):386–. doi: 10.1182/blood.V124.21.386.386

[142] Jiang X Mak PY Mu H Tao W Mak DH Kornblau S. Disruption of Wnt/B-catenin exerts antileukemia activity and synergizes with Flt3 inhibition in Flt3-mutant acute myeloid leukemia. Clin Cancer Res (2018) 24(10):2417–29. doi: 10.1158/1078-0432.Ccr-17-1556 PMC5955840 29463558

[143] Ma S Yang LL Niu T Cheng C Zhong L Zheng MW. Sklb-677, an Flt3 and Wnt/B-catenin signaling inhibitor, displays potent activity in models of Flt3-driven aml. Sci Rep (2015) 5:15646. doi: 10.1038/srep15646 26497577 PMC4620497

[144] Fiskus W Sharma S Saha S Shah B Devaraj SG Sun B. Pre-clinical efficacy of combined therapy with novel B-catenin antagonist Bc2059 and histone deacetylase inhibitor against aml cells. Leukemia (2015) 29(6):1267–78. doi: 10.1038/leu.2014.340 PMC4456205 25482131

[145] Bae MH Oh SH Park CJ Lee BR Kim YJ Cho YU. Vla-4 and Cxcr4 expression levels show contrasting prognostic impact (Favorable and unfavorable, respectively) in acute myeloid leukemia. Ann Hematol (2015) 94(10):1631–8. doi: 10.1007/s00277-015-2442-8 26155911

[146] Hsieh Y-T Jiang E Pham J Kim H-N Abdel-Azim H Khazal S. Vla4 blockade in acute myeloid leukemia. Blood (2013) 122(21):3944–. doi: 10.1182/blood.V122.21.3944.3944

[147] Matsunaga T Fukai F Miura S Nakane Y Owaki T Kodama H. Combination therapy of an anticancer drug with the Fniii14 peptide of fibronectin effectively overcomes cell adhesion-mediated drug resistance of acute myelogenous leukemia. Leukemia (2008) 22(2):353–60. doi: 10.1038/sj.leu.2405017 17972943

[148] Gutjahr JC Bayer E Yu X Laufer JM Höpner JP Tesanovic S. Cd44 engagement enhances acute myeloid leukemia cell adhesion to the bone marrow microenvironment by increasing vla-4 avidity. Haematologica (2021) 106(8):2102–13. doi: 10.3324/haematol.2019.231944 PMC8327716 32616529

[149] Vey N Delaunay J Martinelli G Fiedler W Raffoux E Prebet T. Phase I clinical study of Rg7356, an anti-Cd44 humanized antibody, in patients with acute myeloid leukemia. Oncotarget (2016) 7(22):32532–42. doi: 10.18632/oncotarget.8687 PMC5078031 27081038

[150] Barbier V Erbani J Fiveash C Davies JM Tay J Tallack MR. Endothelial e-selectin inhibition improves acute myeloid leukaemia therapy by disrupting vascular niche-mediated chemoresistance. Nat Commun (2020) 11(1):2042. doi: 10.1038/s41467-020-15817-5 32341362 PMC7184728

[151] Tettamanti S Pievani A Biondi A Dotti G Serafini M. Catch me if you can: How aml and its niche escape immunotherapy. Leukemia (2022) 36(1):13–22. doi: 10.1038/s41375-021-01350-x 34302116 PMC8727297

[152] Lopez-Yrigoyen M Cassetta L Pollard JW. Macrophage targeting in cancer. Ann N Y Acad Sci (2021) 1499(1):18–41. doi: 10.1111/nyas.14377 32445205

[153] Lamble AJ Lind EF. Targeting the immune microenvironment in acute myeloid leukemia: A focus on T cell immunity. Front Oncol (2018) 8:213. doi: 10.3389/fonc.2018.00213 29951373 PMC6008423

[154] Tamura H Dan K Tamada K Nakamura K Shioi Y Hyodo H. Expression of functional B7-H2 and B7.2 costimulatory molecules and their prognostic implications in *De novo* acute myeloid leukemia. Clin Cancer Res (2005) 11(16):5708–17. doi: 10.1158/1078-0432.Ccr-04-2672 16115907

[155] Krönig H Kremmler L Haller B Englert C Peschel C Andreesen R. Interferon-induced programmed death-ligand 1 (Pd-L1/B7-H1) expression increases on human acute myeloid leukemia blast cells during treatment. Eur J Haematol (2014) 92(3):195–203. doi: 10.1111/ejh.12228 24175978

[156] Gonçalves Silva I Yasinska IM Sakhnevych SS Fiedler W Wellbrock J Bardelli M. The Tim-3-Galectin-9 secretory pathway is involved in the immune escape of human acute myeloid leukemia cells. EBioMedicine (2017) 22:44–57. doi: 10.1016/j.ebiom.2017.07.018 28750861 PMC5552242

[157] Almeida-Porada G Ascensāo JL. Isolation, characterization, and biologic features of bone marrow endothelial cells. J Lab Clin Med (1996) 128(4):399–407. doi: 10.1016/S0022-2143(96)80012-6 8833889

[158] Balamurugan K. Hif-1 at the crossroads of hypoxia, inflammation, and cancer. Int J Cancer (2016) 138(5):1058–66. doi: 10.1002/ijc.29519 PMC4573780 25784597

[159] Liu Y Shaw SK Ma S Yang L Luscinskas FW Parkos CA. Regulation of leukocyte transmigration: Cell surface interactions and signaling events. J Immunol (2004) 172(1):7–13. doi: 10.4049/jimmunol.172.1.7 14688302

[160] Huang Y Kim BYS Chan CK Hahn SM Weissman IL Jiang W. Improving immune-vascular crosstalk for cancer immunotherapy. Nat Rev Immunol (2018) 18(3):195–203. doi: 10.1038/nri.2017.145 29332937 PMC5922422

[161] Khan KA Kerbel RS. Improving immunotherapy outcomes with anti-angiogenic treatments and vice versa. Nat Rev Clin Oncol (2018) 15(5):310–24. doi: 10.1038/nrclinonc.2018.9 29434333

[162] Limagne E Richard C Thibaudin M Fumet JD Truntzer C Lagrange A. Tim-3/Galectin-9 pathway and mmdsc control primary and secondary resistances to pd-1 blockade in lung cancer patients. Oncoimmunology (2019) 8(4):e1564505. doi: 10.1080/2162402x.2018.1564505 30906658 PMC6422400

[163] van Galen P Hovestadt V Wadsworth Ii MH Hughes TK Griffin GK Battaglia S. Single-cell rna-seq reveals aml hierarchies relevant to disease progression and immunity. Cell (2019) 176(6):1265–81.e24. doi: 10.1016/j.cell.2019.01.031 30827681 PMC6515904

[164] Williams P Basu S Garcia-Manero G Hourigan CS Oetjen KA Cortes JE. The distribution of T-cell subsets and the expression of immune checkpoint receptors and ligands in patients with newly diagnosed and relapsed acute myeloid leukemia. Cancer (2019) 125(9):1470–81. doi: 10.1002/cncr.31896 PMC6467779 30500073

[165] Shi Y Liu Z Wang H. Expression of pd-L1 on regulatory b cells in patients with acute myeloid leukaemia and its effect on prognosis. J Cell Mol Med (2022) 26(12):3506–12. doi: 10.1111/jcmm.17390 PMC9189343 35610758

[166] Kang CW Dutta A Chang LY Mahalingam J Lin YC Chiang JM. Apoptosis of tumor infiltrating effector Tim-3+Cd8+ T cells in colon cancer. Sci Rep (2015) 5:15659. doi: 10.1038/srep15659 26493689 PMC4616166

[167] Zhu C Anderson AC Schubart A Xiong H Imitola J Khoury SJ. The Tim-3 ligand galectin-9 negatively regulates T helper type 1 immunity. Nat Immunol (2005) 6(12):1245–52. doi: 10.1038/ni1271 16286920

[168] Noviello M Manfredi F Ruggiero E Perini T Oliveira G Cortesi F. Bone marrow central memory and memory stem T-cell exhaustion in aml patients relapsing after hsct. Nat Commun (2019) 10(1):1065. doi: 10.1038/s41467-019-08871-1 30911002 PMC6434052

[169] Deng M Gui X Kim J Xie L Chen W Li Z. Lilrb4 signalling in leukaemia cells mediates T cell suppression and tumour infiltration. Nature (2018) 562(7728):605–9. doi: 10.1038/s41586-018-0615-z PMC6296374 30333625

[170] Coles SJ Hills RK Wang EC Burnett AK Man S Darley RL. Increased Cd200 expression in acute myeloid leukemia is linked with an increased frequency of Foxp3+ regulatory T cells. Leukemia (2012) 26(9):2146–8. doi: 10.1038/leu.2012.75 PMC3460214 22430636

[171] Lv Y Wang H Liu Z. The role of regulatory b cells in patients with acute myeloid leukemia. Med Sci Monit (2019) 25:3026–31. doi: 10.12659/msm.915556 PMC6496973 31017878

[172] Ren J Lan T Liu T Liu Y Shao B Men K. Cxcl13 as a novel immune checkpoint for regulatory b cells and its role in tumor metastasis. J Immunol (2022) 208(10):2425–35. doi: 10.4049/jimmunol.2100341 PMC9125199 35437281

[173] Hasan MM Nair SS O’Leary JG Thompson-Snipes L Nyarige V Wang J. Implication of tigit(+) human memory b cells in immune regulation. Nat Commun (2021) 12(1):1534. doi: 10.1038/s41467-021-21413-y 33750787 PMC7943800

[174] Sun H Li Y Zhang ZF Ju Y Li L Zhang BC. Increase in myeloid-derived suppressor cells (Mdscs) associated with minimal residual disease (Mrd) detection in adult acute myeloid leukemia. Int J Hematol (2015) 102(5):579–86. doi: 10.1007/s12185-015-1865-2 26358057

[175] Yu S Ren X Li L. Myeloid-derived suppressor cells in hematologic malignancies: Two sides of the same coin. Exp Hematol Oncol (2022) 11(1):43. doi: 10.1186/s40164-022-00296-9 35854339 PMC9295421

[176] Peranzoni E Ingangi V Masetto E Pinton L Marigo I. Myeloid cells as clinical biomarkers for immune checkpoint blockade. Front Immunol (2020) 11:1590. doi: 10.3389/fimmu.2020.01590 32793228 PMC7393010

[177] Pyzer AR Stroopinsky D Rajabi H Washington A Tagde A Coll M. Muc1-mediated induction of myeloid-derived suppressor cells in patients with acute myeloid leukemia. Blood (2017) 129(13):1791–801. doi: 10.1182/blood-2016-07-730614 PMC5813734 28126925

[178] Hwang HS Han AR Lee JY Park GS Min WS Kim HJ. Enhanced anti-leukemic effects through induction of immunomodulating microenvironment by blocking Cxcr4 and pd-L1 in an aml mouse model. Immunol Invest (2019) 48(1):96–105. doi: 10.1080/08820139.2018.1497057 30204524

[179] Mantovani A Sozzani S Locati M Allavena P Sica A. Macrophage polarization: Tumor-associated macrophages as a paradigm for polarized M2 mononuclear phagocytes. Trends Immunol (2002) 23(11):549–55. doi: 10.1016/s1471-4906(02)02302-5 12401408

[180] Miari KE Guzman ML Wheadon H Williams MTS. Macrophages in acute myeloid leukaemia: Significant players in therapy resistance and patient outcomes. Front Cell Dev Biol (2021) 9:692800. doi: 10.3389/fcell.2021.692800 34249942 PMC8264427

[181] Al-Matary YS Botezatu L Opalka B Hönes JM Lams RF Thivakaran A. Acute myeloid leukemia cells polarize macrophages towards a leukemia supporting state in a growth factor independence 1 dependent manner. Haematologica (2016) 101(10):1216–27. doi: 10.3324/haematol.2016.143180 PMC5046651 27390361

[182] Majeti R Chao MP Alizadeh AA Pang WW Jaiswal S Gibbs KD Jr.. Cd47 is an adverse prognostic factor and therapeutic antibody target on human acute myeloid leukemia stem cells. Cell (2009) 138(2):286–99. doi: 10.1016/j.cell.2009.05.045 PMC2726837 19632179

[183] Study evaluating the safety and effectiveness magrolimab versus placebo in combination with venetoclax and azacitidine in participants with acute myeloid leukemia (Aml) (Enhance-3) (2022). Available at: https://clinicaltrials.gov/ct2/show/NCT05079230.

[184] Liu J Wang L Zhao F Tseng S Narayanan C Shura L. Pre-clinical development of a humanized anti-Cd47 antibody with anti-cancer therapeutic potential. PloS One (2015) 10(9):e0137345. doi: 10.1371/journal.pone.0137345 26390038 PMC4577081

[185] Barrett AJ Le Blanc K. Immunotherapy prospects for acute myeloid leukaemia. Clin Exp Immunol (2010) 161(2):223–32. doi: 10.1111/j.1365-2249.2010.04197.x PMC2909404 20529084

[186] Dai YJ He SY Hu F Li XP Zhang JM Chen SL. Bone marrow infiltrated natural killer cells predicted the anti-leukemia activity of Mcl1 or Bcl2 inhibitors in acute myeloid leukemia. Mol Cancer (2021) 20(1):8. doi: 10.1186/s12943-020-01302-6 33402171 PMC7784307

[187] Rahmani S Yazdanpanah N Rezaei N. Natural killer cells and acute myeloid leukemia: Promises and challenges. Cancer Immunol Immunother (2022) 71(12):2849–67. doi: 10.1007/s00262-022-03217-1 PMC10991240 35639116

[188] Fauriat C Just-Landi S Mallet F Arnoulet C Sainty D Olive D. Deficient expression of ncr in nk cells from acute myeloid leukemia: Evolution during leukemia treatment and impact of leukemia cells in ncrdull phenotype induction. Blood (2007) 109(1):323–30. doi: 10.1182/blood-2005-08-027979 16940427

[189] Mastaglio S Wong E Perera T Ripley J Blombery P Smyth MJ. Natural killer receptor ligand expression on acute myeloid leukemia impacts survival and relapse after chemotherapy. Blood Adv (2018) 2(4):335–46. doi: 10.1182/bloodadvances.2017015230 PMC5858482 29449224

[190] Hsu J Hodgins JJ Marathe M Nicolai CJ Bourgeois-Daigneault MC Trevino TN. Contribution of nk cells to immunotherapy mediated by pd-1/Pd-L1 blockade. J Clin Invest (2018) 128(10):4654–68. doi: 10.1172/jci99317 PMC6159991 30198904

[191] Lanuza PM Pesini C Arias MA Calvo C Ramirez-Labrada A Pardo J. Recalling the biological significance of immune checkpoints on nk cells: A chance to overcome Lag3, Pd1, and Ctla4 inhibitory pathways by adoptive nk cell transfer? Front Immunol (2019) 10:3010. doi: 10.3389/fimmu.2019.03010 31998304 PMC6962251

[192] Khan M Arooj S Wang H. Nk cell-based immune checkpoint inhibition. Front Immunol (2020) 11:167. doi: 10.3389/fimmu.2020.00167 32117298 PMC7031489

[193] Razazian M Khosravi M Bahiraii S Uzan G Shamdani S Naserian S. Differences and similarities between mesenchymal stem cell and endothelial progenitor cell immunoregulatory properties against T cells. World J Stem Cells (2021) 13(8):971–84. doi: 10.4252/wjsc.v13.i8.971 PMC8422932 34567420

[194] Tan Z Kan C Wong M Sun M Liu Y Yang F. Regulation of malignant myeloid leukemia by mesenchymal stem cells. Front Cell Dev Biol (2022) 10:857045. doi: 10.3389/fcell.2022.857045 35756991 PMC9213747

[195] Davies LC Heldring N Kadri N Le Blanc K. Mesenchymal stromal cell secretion of programmed death-1 ligands regulates T cell mediated immunosuppression. Stem Cells (2017) 35(3):766–76. doi: 10.1002/stem.2509 PMC5599995 27671847

[196] Ambrosi TH Scialdone A Graja A Gohlke S Jank AM Bocian C. Adipocyte accumulation in the bone marrow during obesity and aging impairs stem cell-based hematopoietic and bone regeneration. Cell Stem Cell (2017) 20(6):771–84.e6. doi: 10.1016/j.stem.2017.02.009 28330582 PMC5459794

[197] Yue R Zhou BO Shimada IS Zhao Z Morrison SJ. Leptin receptor promotes adipogenesis and reduces osteogenesis by regulating mesenchymal stromal cells in adult bone marrow. Cell Stem Cell (2016) 18(6):782–96. doi: 10.1016/j.stem.2016.02.015 27053299

[198] Miggitsch C Meryk A Naismith E Pangrazzi L Ejaz A Jenewein B. Human bone marrow adipocytes display distinct immune regulatory properties. EBioMedicine (2019) 46:387–98. doi: 10.1016/j.ebiom.2019.07.023 PMC6711052 31327694

[199] Horowitz MC Berry R Holtrup B Sebo Z Nelson T Fretz JA. Bone marrow adipocytes. Adipocyte (2017) 6(3):193–204. doi: 10.1080/21623945.2017.1367881 28872979 PMC5638373

[200] Wu B Sun X Gupta HB Yuan B Li J Ge F. Adipose pd-L1 modulates pd-1/Pd-L1 checkpoint blockade immunotherapy efficacy in breast cancer. Oncoimmunology (2018) 7(11):e1500107. doi: 10.1080/2162402x.2018.1500107 30393583 PMC6209395

[201] Picarda E Galbo PM Jr. Zong H Rajan MR Wallenius V Zheng D. The immune checkpoint B7-H3 (Cd276) regulates adipocyte progenitor metabolism and obesity development. Sci Adv (2022) 8(17):eabm7012. doi: 10.1126/sciadv.abm7012 35476450 PMC9045715

[202] Menter T Tzankov A. Tumor microenvironment in acute myeloid leukemia: Adjusting niches. Front Immunol (2022) 13:811144. doi: 10.3389/fimmu.2022.811144 35273598 PMC8901718

[203] Tang T Huang X Zhang G Hong Z Bai X Liang T. Advantages of targeting the tumor immune microenvironment over blocking immune checkpoint in cancer immunotherapy. Signal Transduct Target Ther (2021) 6(1):72. doi: 10.1038/s41392-020-00449-4 33608497 PMC7896069

[204] Zeng Y Li B Liang Y Reeves PM Qu X Ran C. Dual blockade of Cxcl12-Cxcr4 and pd-1-Pd-L1 pathways prolongs survival of ovarian tumor-bearing mice by prevention of immunosuppression in the tumor microenvironment. FASEB J (2019) 33(5):6596–608. doi: 10.1096/fj.201802067RR PMC6463916 30802149

[205] Huang KC Chiang SF Chen WT Chen TW Hu CH Yang PC. Decitabine augments chemotherapy-induced pd-L1 upregulation for pd-L1 blockade in colorectal cancer. Cancers (Basel) (2020) 12(2):462. doi: 10.3390/cancers12020462 32079180 PMC7072566

